# Functional Analysis of Cellulose Synthase *CesA4* and *CesA6* Genes in Switchgrass (*Panicum virgatum*) by Overexpression and RNAi-Mediated Gene Silencing

**DOI:** 10.3389/fpls.2018.01114

**Published:** 2018-08-03

**Authors:** Mitra Mazarei, Holly L. Baxter, Mi Li, Ajaya K. Biswal, Keonhee Kim, Xianzhi Meng, Yunqiao Pu, Wegi A. Wuddineh, Ji-Yi Zhang, Geoffrey B. Turner, Robert W. Sykes, Mark F. Davis, Michael K. Udvardi, Zeng-Yu Wang, Debra Mohnen, Arthur J. Ragauskas, Nicole Labbé, C. Neal Stewart

**Affiliations:** ^1^Department of Plant Sciences, University of Tennessee, Knoxville, Knoxville, TN, United States; ^2^BioEnergy Science Center, Oak Ridge National Laboratory, Oak Ridge, TN, United States; ^3^Biosciences Division, Joint Institute for Biological Science, Oak Ridge National Laboratory, Oak Ridge, TN, United States; ^4^Complex Carbohydrate Research Center, University of Georgia, Athens, GA, United States; ^5^Center for Renewable Carbon, University of Tennessee, Knoxville, Knoxville, TN, United States; ^6^Department of Chemical and Biomolecular Engineering, University of Tennessee, Knoxville, Knoxville, TN, United States; ^7^Noble Research Institute, Ardmore, OK, United States; ^8^National Renewable Energy Laboratory, Golden, CO, United States

**Keywords:** cellulose synthase, switchgrass, overexpression, RNAi-gene silencing, *PvCesA4*, *PvCesA6*, lignocellulosic, biofuel

## Abstract

Switchgrass (*Panicum virgatum* L.) is a leading lignocellulosic bioenergy feedstock. Cellulose is a major component of the plant cell walls and the primary substrate for saccharification. Accessibility of cellulose to enzymatic breakdown into fermentable sugars is limited by the presence of lignin in the plant cell wall. In this study, putatively novel switchgrass secondary cell wall cellulose synthase *PvCesA4* and primary cell wall *PvCesA6* genes were identified and their functional role in cellulose synthesis and cell wall composition was examined by overexpression and knockdown of the individual genes in switchgrass. The endogenous expression of *PvCesA4* and *PvCesA6* genes varied among including roots, leaves, stem, and reproductive tissues. Increasing or decreasing *PvCesA4* and *PvCesA6* expression to extreme levels in the transgenic lines resulted in decreased biomass production. *PvCesA6*-overexpressing lines had reduced lignin content and syringyl/guaiacyl lignin monomer ratio accompanied by increased sugar release efficiency, suggesting an impact of *PvCesA6* expression levels on lignin biosynthesis. Cellulose content and cellulose crystallinity were decreased, while xylan content was increased in *PvCesA4* and *PvCesA6* overexpression or knockdown lines. The increase in xylan content suggests that the amount of non-cellulosic cell wall polysaccharide was modified in these plants. Taken together, the results show that the manipulation of the cellulose synthase genes alters the cell wall composition and availability of cellulose as a bioprocessing substrate.

## Introduction

Plant cell walls consist largely of polysaccharides (cellulose, hemicellulose, pectin) and the polyphenolic compound lignin (Somerville et al., 2004). Cellulose is the most abundant constituent of primary and secondary cell walls in plants and plays a central role in plant mechanical strength and morphogenesis (Cosgrove, 2005; Liu et al., 2016). Cellulose is made up of chains containing repeated glucose residues, which together form strong microfibril structures (Somerville, 2006). In higher plants, cellulose is synthesized by a large cellulose synthase (CesA) complex located on the plasma membrane (Schneider et al., 2016). Since the first plant *CesA* gene was identified from cotton (Pear et al., 1996), the CesA superfamily has been characterized in many plant species, including Arabidopsis (Taylor et al., 2003; Desprez et al., 2007; Persson et al., 2007), rice (Tanaka et al., 2003; Wang et al., 2010), maize (Appenzeller et al., 2004), cotton (Li A. et al., 2013), barley (Burton et al., 2004), and poplar (Joshi et al., 2004; Djerbi et al., 2005). While cellulose biosynthesis is not fully understood, work with Arabidopsis mutants has elucidated the key genes encoding the catalytic subunits of CesA, with some involved in making primary cell walls (AtCesA1, AtCesA3, AtCesA6) and others in making secondary cell walls (AtCesA4, AtCesA7, AtCesA8) (Endler and Persson, 2011). At least three CesAs each are expressed in cells during either primary or secondary wall formation, and mutations in any one of them disrupt cellulose synthesis, indicating the non-redundant function of members of the different subclass members (Somerville, 2006).

Switchgrass (*Panicum virgatum* L.) is a promising lignocellulosic bioenergy feedstock owing to its wide adaptation, high genetic variability, and its ability to reliably produce easily-harvested aboveground biomass each year. The resistance of plant cell walls to deconstruction, defined as biomass recalcitrance, hinders the accessibility of cellulose to enzymatic breakdown into fermentable sugars for biofuel production (Himmel and Bayer, 2009). Biomass recalcitrance is mainly determined by the cell wall composition and its complex structure. Lignin is the primary contributor to biomass recalcitrance (Chen and Dixon, 2007). Genetic engineering of plant cell walls has been shown to reduce biomass recalcitrance (Nelson et al., 2017; Biswal et al., 2018). Since cellulose is a major structural component of the cell wall and the primary substrate for saccharification, manipulating the genes involved in cellulose synthesis could alter the cell wall composition and availability of cellulose as a bioprocessing substrate (Mizrachi et al., 2012; Kalluri et al., 2014; Bali et al., 2016). The practical interest of such an investigation for switchgrass is supported by studies demonstrating that a functional relationship exists between CesA structures, cellulose crystallinity and saccharification efficiency in Arabidopsis and rice (Harris et al., 2012; Li F. et al., 2017).

In the present study, novel switchgrass cellulose synthase *PvCesA4* and *PvCesA6* genes were identified and their functional role in cellulose synthesis was examined by overexpression and knockdown of the individual genes in switchgrass. These transgenic plants were analyzed for (i) *PvCesA* expression, (ii) growth morphology and biomass yield, (iii) cell wall composition and properties, and (iv) sugar release efficiency. To our knowledge, this is the first functional description of switchgrass CesA genes.

## Materials and Methods

### Gene Identification

Using the CesA amino acid sequences of Arabidopsis (*Arabidopsis thaliana*), rice (*Oryza sativa*), and maize (*Zea mays*) as heterologous probes, TBLASTN was used to identify the homologous gene sequences from switchgrass EST databases (Zhang J.Y. et al., 2013) and the draft genome (*Panicum virgatum* v1.1 DOE-JGI) at Phytozome. A phylogenetic tree of CesAs protein family members of *A. thaliana* TAIR10, *O. sativa* v7.0, *Populus trichocarpa* v3.0, *Setaria italica* v2.2, and *P. virgatum* v1.1 from Phytozome 12.0^1^ was constructed by the neighbor-joining method using MEGA6 (Tamura et al., 2013). The CesA phylogenetic tree was divided into 6-clades [Clade A (CESA1), Clade B (CESA3), Clade C (CESA6), Clade D (CESA7), Clade E (CESA8), and Clade F (CESA4)] based on Kumar et al. (2009) and Kumar and Turner (2015). Pavir.Ib00804 from Clade F and Pavir.Ba01088 from Clade C were named PvCesA4 and PvCesA6, respectively.

### Vector Construction and Transgenic Plant Production

Overexpression cassettes were constructed by isolating the gene open reading frame (ORF) from switchgrass cDNAs of the ST1 clonal genotype of “Alamo” switchgrass using individual gene-specific primers flanking the ORF of each gene (Supplementary Figures S1, S2 and Supplementary Table S1) and subsequently cloning each into pCR8 entry vector for sequence confirmation. For RNAi cassettes, target sequences of 223 bp (*PvCesA4*) and 331 bp (*PvCesA6*) were used (Supplementary Figures S1, S2) and cloned into pCR8 vector for sequence confirmation. Sequence-confirmed fragments were then sub-cloned into pANIC-10A overexpression vector or into pANIC-8A RNAi-vector (Supplementary Figure S3) by GATEWAY recombination (Mann et al., 2012) to place each target gene under the control of the maize ubiquitin 1 (*ZmUbi1*) promoter. Embryogenic callus derived from “Alamo” switchgrass NFCX01 genotype was transformed with the expression vector construct using *Agrobacterium*-mediated transformation (Xi et al., 2009).

### Plants and Growth Conditions

Transgenic and non-transgenic control plants were grown under the same conditions (16 h day/8 h night light at 26°C) in growth chambers. For growth analysis, each transgenic and non-transgenic line was propagated from a single tiller to yield three clonal replicates each (Hardin et al., 2013). The growth parameters were measured at R1 growth stage (Moore et al., 1991). Plant height was determined by measuring the five tallest tillers from each replicate. Stem diameters were measured for each of these tillers with a digital caliper. For plant width, the diameter of the plant crown mid-section was measured. Tiller numbers were tallied for each plant. The fresh biomass was measured from the aboveground plant biomass cut at a similar stage of growth while the dry biomass was measured from fresh biomass dried at 42°C for 96 h.

### RNA Extraction and qRT-PCR

Total RNA was extracted from root, leaf sheath, leaf blade, stem, and panicle samples at the R1 growth stage or from the shoot tips of transgenic lines at the E4 growth stage using Tri-Reagent (Sigma-Aldrich, St. Louis, MO, United States) following the manufacturer’s instructions. One microgram of the purified RNA was treated with DNase-1 (Qiagen, Valencia, CA, United States) to remove any potential genomic DNA contaminants. The DNase-treated RNA was used for first-strand cDNA synthesis using High-Capacity cDNA Reverse Transcription kit (Applied Biosystems, Foster City, CA, United States). qRT-PCR experiments were performed with Power SYBR Green PCR Master Mix (Applied Biosystems) in an optical 96-well plate using a Quant Studio 6 Flex system (Applied Biosystems). Analysis of the relative expression was carried out by the change in Ct method. The standard curve method was used for relative transcript quantification normalized by switchgrass ubiquitin 1 (*PvUbi1*) as a reference gene (Shen et al., 2009). Primers used for transcript analysis are listed in Supplementary Table S1.

### Cell Wall Characterization

Tillers were collected at the R1 growth stage, dried at 42°C for 96 h, and ground to 0.5 mm (40 mesh) particle size. Cell wall chemical composition was determined following the National Renewable Energy Laboratory (NREL) protocols. Briefly, approximately 3 g samples were sequentially extracted with water and ethanol using an automated extraction system (ASE 350, Dionex Corp., Sunnyvale, CA, United States) following the NREL protocol “Determination of extractives in biomass (NREL/TP 510-42619).” The extracted samples were then dried at 40°C for 3 days until constant weight. Cellulose, hemicellulose, lignin, acetyl content, and structural ash were then measured after a two-step acid hydrolysis following the NREL protocol “Determination of structural carbohydrates and lignin in biomass (NREL/TP 510-42618).” High pressure liquid chromatography (HPLC) was employed to quantify the structural monomeric sugars after the two-step of acid hydrolysis. Acid insoluble lignin was measured gravimetrically and acid soluble lignin was measured using a Genesys 10S UV-Vis Spectrophotometer (Thermo Scientific, Dubuque, IA, United States). The HPLC system for carbohydrates measurement was equipped with an Aminex HPX-87P column (300 nm × 7.8 nm, 9 μm particle sizes) (Bio-Rad, Hercules, CA, United States) attached to a micro-guard Carbo-P guard column (Bio-Rad), and a refractive index detector (Perkin Elmer, Waltham, MA, United States). The HPLC’s RI detector temperature was 50°C and the oven temperature was set at 85°C. The injection volume was 30 μl with 0.25 ml/min of flow rate. Mannose peak was not detected in this study, and the total hemicellulose content was the sum of xylose, galactose, and arabinose. The acetyl content was also measured utilizing HPLC equipped with Aminex HPX-87H column (300 nm × 7.8 nm, 9 μm particle size) (Bio-Rad). The RI detector temperature was 50°C and the oven temperature was set at 45°C. The mobile phase was 0.05 M sulfuric acid. The injection volume was 50 μl with 0.6 ml/min of flow rate. The total (unextracted biomass) and structural ash (extractives-free biomass) content was gravimetrically determined by combusting 0.5 g of biomass in a furnace (Fisher Scientific Isotemp Programmable Muffle Furnace 750, Dubuque, IA, United States) at 575°C for 24 h following the NREL protocol “Determination of ash in biomass (NREL/TP 510-42622).” Lignin composition was determined by pyrolysis molecular beam mass spectrometry (py-MBMS) using the NREL high-throughput method on extractive- and starch-free samples (Sykes et al., 2009; Decker et al., 2012). Sugar release by enzymatic hydrolysis was determined by NREL high-throughput method on extractive- and starch-free samples described by Selig et al. (2010). Briefly, cell wall residues were prepared by removing soluble extractives and starch. Samples were loaded into a 96-well plate. A hot water pretreatment was conducted in a steam chamber at 180°C for 17.5 min. After pretreatment, enzymatic hydrolysis was performed in the well plate on the pretreated slurry by incubation with Ctec2 enzyme cocktail (70 mg protein/g biomass) at 40°C for 72 h. Glucose and xylose release were determined by colorimetric assays, and total sugar release is the sum of glucose + xylose released (Studer et al., 2009).

### Cellulose Characterization

Cellulose properties were determined using tillers collected at the R1 growth stage and dried at 42°C for 96 h, followed by milling to 1 mm (20 mesh) particle size. Cellulose isolation and the measurements of cellulose crystallinity and its degree of polymerization were conducted as previously described (Li M. et al., 2017). Briefly, the extractives of switchgrass were removed by extraction using ethanol:toluene mixture (1:2, v:v) for 24 h. The extractives-free switchgrass samples were delignified by peracetic acid (32% solution in acetic acid) and air-dried to obtain switchgrass holocellulose. One portion of the holocellulose was subjected to hydrochloric acid in boiling water bath to remove hemicellulose. The residue cellulose was washed with deionized water, filtered, and used to measure cellulose crystallinity using cross polarization magic angle spinning (CP/MAS) on a Bruker Avance-400 spectrometer. The cellulose crystallinity index (CrI) was determined from the areas of the crystalline and amorphous C_4_ signals of cellulose. Another portion of the holocellulose was extracted with sodium hydroxide. The obtained cellulose residue, namely α-cellulose, was used to measure the weight-average molecular weight (M_w_) and number-average molecular weight (M_n_) of cellulose using gel permeation chromatographic analysis after tricarbanilation. The weight-average (DP_w_) and number-average (DP_n_) degree of polymerization of cellulose were calculated by dividing the M_w_ and M_n_ by 519 g/mol, the molecular mass of repeating unit of derivatized cellulose. Both the cellulose crystallinity and its degree of polymerization were reported as the average value of three biological replicates.

### Statistics

Statistical analyses were performed in SAS version 9.4 (SAS Institute Inc., Cary, NC, United States). One-way ANOVA with Fisher’s least significant difference method was used to compare means among transgenic lines and the control. Differences were considered significant when *P*-values were less than or equal to 0.05. For pairwise comparisons, each transgenic line was compared with the control using the PROC TTEST procedure in SAS. Differences were considered significant when *P*-values were less than or equal to 0.05.

## Results

### Identification of PvCesA Homologs

**FIGURE 1:**
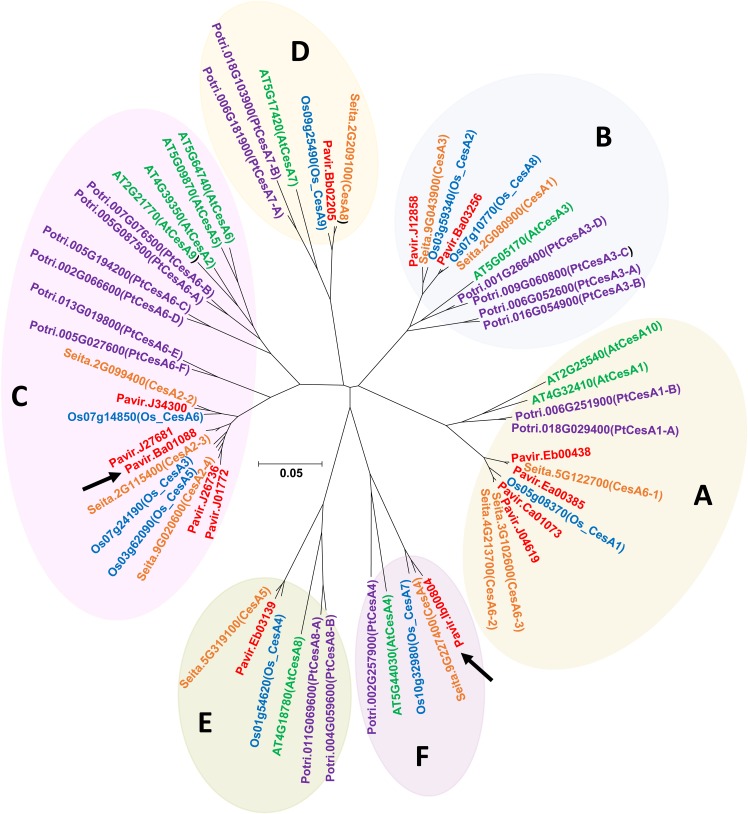
The gene tree of the CesA protein family. Various members are shown by plant species: *Arabidopsis thaliana* TAIR10 (green), *Populus trichocarpa* v3.0 (purple), *O. sativa* v7.0 (blue), *Setaria italica* (orange), and *Panicum virgatum* v1.1 (red) from Phytozome 12.0 (https://phytozome.jgi.doe.gov/) showing relationship between amino acid sequences. The CesA phylogenetic tree was divided into 6-clades [Clade **A** (CESA1), Clade **B** (CESA3), Clade **C** (CESA6), Clade **D** (CESA7), Clade **E** (CESA8), and Clade **F** (CESA4)] based on Kumar and Turner (2015) and Kumar et al., 2009. Pavir.Ib00804 and Pavir.Ba01088 (marked by black arrows) were named CesA4 and CesA6 for *P. virgatum*, respectively.

The CesA orthologous amino acid sequences from Arabidopsis, rice, and maize were used to identify the switchgrass PvCesA sequences and CesA protein family members of *A. thaliana*, *P. trichocarpa*, *O. sativa*, *S. italica*, and *P. virgatum* from Phytozome were used to elucidate amino acid sequences profiles (**Figure 1**). Based on this, Pavir.Ib00804 from Clade F and Pavir.Ba01088 from Clade C were identified and named PvCesA4 and PvCesA6 for *P. virgatum*, respectively (**Figure 1**). At the time that this work was started, Pavir.Ib00804 and Pavir.Ba01088 were indicated as the genes for switchgrass CesA4 and CesA6, respectively. After completion and release of the switchgrass genome, four other related CesAs were located in the Clade C (CESA6) (**Figure 1**).

### Expression Patterns of *PvCesA4* and *PvCesA6* in Non-transgenic Switchgrass

**FIGURE 2:**
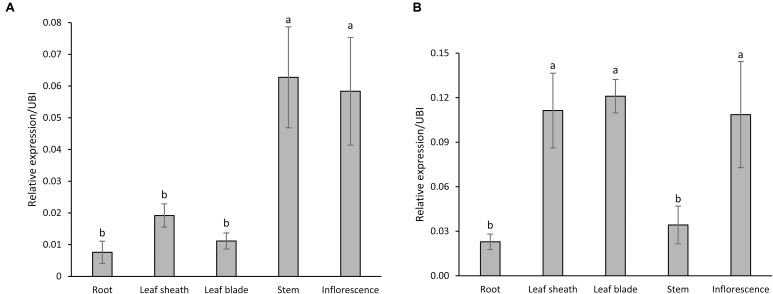
Expression patterns of *PvCesA4* **(A)** and *PvCesA6* **(B)** in different plant tissues as determined by qRT-PCR. Plant samples for RNA extraction used in the qRT-PCR experiments were collected at R1 (reproductive stage 1) developmental stage. The relative levels of transcripts were normalized to the switchgrass ubiquitin 1 gene expression (UBI). Bars represent mean values of three biological replicates ± standard error. Bars represented by different letters are significantly different at *P* ≤ 0.05 as tested by LSD method with SAS software (SAS Institute Inc.).

Quantitative reverse transcription-polymerase chain reaction (qRT-PCR) results revealed detectable levels of expression for both *PvCesA4* and *PvCesA6* genes in roots, leaf sheaths, leaf blades, stems, and inflorescences at the R1 growth stage (**Figures 2A,B**). The expression of *PvCesA4* was highest in stem and inflorescence (**Figure 2A**), whereas the expression of *PvCesA6* was highest in leaves and inflorescence (**Figure 2B**). Furthermore, the expression levels of the other four related *PvCesA6* (Clade C, **Figure 1**) were also highest in leaves (Supplementary Figure S4).

### Generation of Transgenic Plants Overexpressing *PvCesA4* and *PvCesA6*

**FIGURE 3:**
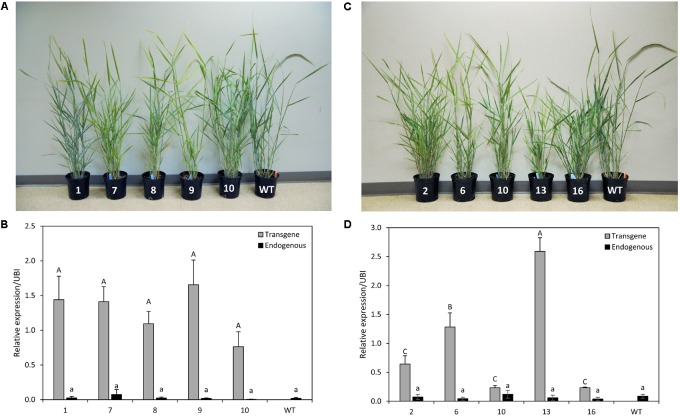
Representative *PvCesA4*-overexpressing **(A)** and *PvCesA6*-overexpressing **(C)** transgenic lines. Relative transcript levels of *PvCesA4* **(B)** and *PvCesA6* **(D)** in overexpressing transgenic lines as determined by qRT-PCR. WT: non-transgenic control. The relative levels of transcripts were normalized to the switchgrass ubiquitin 1 gene expression (UBI). Bars represent mean values of three biological replicates ± standard error. Bars represented by different letters are significantly different at *P* ≤ 0.05 as tested by LSD method with SAS software (SAS Institute Inc.).

Five independent transgenic lines overexpressing either *PvCesA4* or *PvCesA6* driven by the *ZmUbi1* promoter were produced (**Figures 3A,C**). Genomic PCR using primers specific to the transgene and the hygromycin resistance gene confirmed that the plants were stably transgenic (data not shown). *PvCesA4* was overexpressed between 19- and 41-fold in the transgenic lines compared to non-transgenic control by qRT-PCR analysis (**Figure 3B**) and *PvCesA6* was overexpressed between 3- and 30-fold (**Figure 3D**) in the transgenic lines. The level of expression of the endogenous *PvCesA4* and *PvCesA6* was not affected and was similar to the expression levels in non-transgenic control (**Figures 3B,D**).

### Expression Levels of Other Major Secondary and Primary Wall CesAs in Transgenic Plants Overexpressing *PvCesA4* and *PvCesA6*

Transcript abundance of other major secondary wall *PvCesA7* (Clade D) and *PvCesA8* (Clade E), and primary wall *PvCesA1* (Clade A) and *PvCesA3* (Clade B) (**Figure 1**) was determined in *PvCesA4* and *PvCesA6* overexpressing lines. The expression levels of these genes were generally unaffected as compared to those found in non-transgenic control (Supplementary Figure S5).

### Generation of *PvCesA4*-RNAi and *PvCesA6*-RNAi Transgenic Plants

**FIGURE 4:**
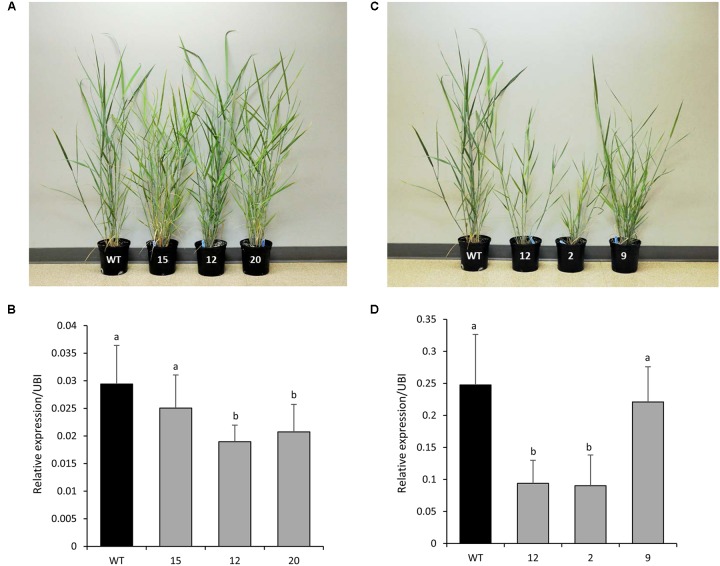
Representative *PvCesA4*-RNAi **(A)** and *PvCesA6*-RNAi **(C)** transgenic lines. Relative transcript levels of *PvCesA4* **(B)** and *PvCesA6* **(D)** in RNAi-transgenic lines as determined by qRT-PCR. WT: non-transgenic control. The relative levels of transcripts were normalized to the switchgrass ubiquitin 1 gene expression (UBI). Bars represent mean values of three biological replicates ± standard error. Bars represented by different letters are significantly different at *P* ≤ 0.05 as tested by LSD method with SAS software (SAS Institute Inc.).

Five independent RNAi-transgenic lines for *PvCesA4* and *PvCesA6* driven by the *ZmUbi1* promoter were produced. Three transgenic lines for each gene that had normal growth rates and reached the R1 growth stage were selected for further characterization (**Figures 4A,C**). Genomic PCR using primers specific to the transgene and the hygromycin resistance gene confirmed that the plants were stably transgenic (data not shown). qRT-PCR analysis showed that the *PvCesA4* transcript level was decreased by 15–36% (**Figure 4B**) and the *PvCesA6* transcript level was decreased by 11–64% (**Figure 4D**) in the RNAi-transgenic lines.

### Phenotypic Characterization of Transgenic Plants

#### *PvCesA4*-Overexpressing Lines

There was a statistically significant decrease in tiller height for three transgenic lines (7, 8, and 9) and in plant width for three transgenic lines (1, 8, and 9) compared with non-transgenic controls. There were no significant differences in stem diameter between the transgenic lines and the non-transgenic controls. Tiller number was significantly increased for two transgenic lines (7 and 10) and decreased for one transgenic line (9). All transgenic lines had equivalent dry biomass relative to the non-transgenic control lines with the exception of line-8 (decreased biomass by 33%) and line-9 (decreased biomass by 58%) (**Table 1A**), congruent to the highest transcript level of the transgene (**Figure 3B**).

**Table 1:** Morphology and biomass yields of transgenic lines overexpressing *PvCesA4* or *PvCesA6* and non-transgenic (WT) controls.

A. *PvCesA4*-overexpression						
Lines	1	7	8	9	10	WT
Height (cm)	128.7 ± 5.9	**116.0 ± 2.0^∗^**	**119.9 ± 1.9^∗^**	**109.4 ± 1.7^∗^**	127.0 ± 1.7	130.7 ± 3.0
Width (cm)	**44.9 ± 1.7^∗∗^**	50.0 ± 3.4	**41.5 ± 0.8^∗∗^**	**33.9 ± 5.2^∗^**	49.1 ± 3.4	58.4 ± 1.5
Stem diameter (mm)	5.6 ± 0.1	5.7 ± 0.3	6.2 ± 0.1	6.1 ± 0.7	5.9 ± 0.1	5.9 ± 0.2
Tiller number	18.0 ± 2.1	**24.7 ± 2.9^∗^**	11.0 ± 1.5	**5.0 ± 2.1^∗^**	**23.0 ± 3.5^∗^**	13.7 ± 0.9
Fresh weight (g)	158.5 ± 8.2	186.3 ± 21.7	**92.4 ± 9.4^∗^**	**65.5 ± 26.9^∗^**	169.3 ± 28.3	140.9 ± 11.2
Dry weight (g)	49.5 ± 4.3	50.2 ± 7.2	**26.4 ± 1.5^∗^**	**16.5 ± 7.0^∗^**	46.1 ± 7.5	39.6 ± 4.2

**B. *PvCesA6*-overexpression**						
**Lines**	**2**	**6**	**10**	**13**	**16**	**WT**

Height (cm)	110.9 ± 0.8	**95.4 ± 15.0^∗^**	119.7 ± 4.1	**76.5 ± 6.2^∗∗^**	**103.6 ± 3.7^∗∗^**	130.7 ± 3.0
Width (cm)	59.3 ± 0.8	**38.9 ± 4.2^∗^**	46.6 ± 5.9	**43.2 ± 1.5^∗∗^**	59.3 ± 1.7	58.4 ± 1.5
Stem diameter (mm)	5.3 ± 0.2	**5.0 ± 0.4^∗^**	6.0 ± 0.1	**4.6 ± 0.0^∗∗^**	5.8 ± 0.2	5.9 ± 0.2
Tiller number	**32.7 ± 1.3^∗∗^**	14.0 ± 5.1	14.0 ± 0.6	11.3 ± 2.6	18.3 ± 3.8	13.7 ± 0.9
Fresh weight (g)	**179.1 ± 0.2^∗^**	**65.7 ± 28.0^∗^**	107.3 ± 0.9	**32.2 ± 6.0^∗∗^**	110.7 ± 27.9	140.9 ± 11.2
Dry weight (g)	**47.1 ± 1.3^∗^**	**16.1 ± 7.5^∗^**	25.8 ± 1.3	**7.6 ± 1.8^∗∗^**	29.2 ± 7.3	39.6 ± 4.2

*Values represent the mean of three biological replicates ± standard error. Bold values with asterisks are significantly different from controls at P ≤ 0.05^∗^ and P ≤ 0.01^∗∗^ as calculated using t-tests for pairwise comparison with SAS software (SAS Institute Inc.).*

#### *PvCesA6*-Overexpressing Lines

Two transgenic lines (6 and 13) had consistent significant decrease in tiller height, plant width, and stem diameter compared with non-transgenic controls. These two transgenic lines (6 and 13) also had significantly decreased dry biomass weight to 59% (line-6) and 81% (line-13) relative to non-transgenic controls (**Table 1B**), corresponding to the highest transcript level of the transgene (**Figure 3D**). There were more tillers in transgenic line-2 resulting in a significant increase in dry biomass (19% more) relative to non-transgenic controls (**Table 1B**).

#### *PvCesA4*-RNAi Lines

There were no significant differences in tiller height and stem diameter between the transgenics and non-transgenic controls. There was a significant decrease in plant width for two transgenic lines (12 and 20) and an increase in tiller number for one transgenic line (15) compared with non-transgenic controls. All transgenic lines had equivalent dry biomass weight relative to the non-transgenic controls (**Table 2A**).

**Table 2:** Morphology and biomass yields of *PvCesA4*-RNAi or *PvCesA6*-RNAi transgenic lines and non-transgenic (WT) controls.

A. *PvCesA4*-RNAi				
Lines	12	15	20	WT
Height (cm)	114.0 ± 15.4	117.7 ± 3.8	120.5 ± 2.2	125.9 ± 7.4
Width (cm)	**35.6 ± 5.3^∗^**	48.3 ± 2.5	**33.9 ± 6.1^∗^**	53.3 ± 2.6
Stem diameter (mm)	5.7 ± 0.1	5.6 ± 0.1	5.6 ± 0.1	5.8 ± 0.3
Tiller number	15.0 ± 2.3	**28.0 ± 1.7^∗∗^**	18.3 ± 2.0	13.3 ± 0.3
Fresh weight (g)	97.8 ± 27.2	155.4 ± 7.5	128.2 ± 25.5	122.1 ± 15.9
Dry weight (g)	21.0 ± 6.4	40.6 ± 2.3	26.2 ± 8.6	33.9 ± 2.2

**B. *PvCesA6*-RNAi**				
**Lines**	**2**	**9**	**12**	**WT**

Height (cm)	**83.3 ± 10.0^∗^**	114.3 ± 5.8	**82.7 ± 11.4^∗^**	125.9 ± 7.4
Width (cm)	**35.6 ± 5.3^∗^**	45.7 ± 2.5	**31.3 ± 4.5^∗^**	53.3 ± 2.6
Stem diameter (mm)	5.2 ± 0.0	4.8 ± 0.2	5.2 ± 0.3	5.8 ± 0.3
Tiller number	10.0 ± 1.2	**24.7 ± 2.7^∗^**	**7.7 ± 1.2^∗^**	13.3 ± 0.3
Fresh weight (g)	**50.7 ± 21.6^∗^**	103.5 ± 22.7	**35.9 ± 6.4^∗∗^**	122.1 ± 15.9
Dry weight (g)	**10.7 ± 4.8^∗^**	30.8 ± 12.9	**7.9 ± 1.3^∗∗^**	33.9 ± 2.2

*Values represent the mean of three biological replicates ± standard error. Bold values with asterisks are significantly different from controls at P ≤ 0.05^∗^ and P ≤ 0.01^∗∗^ as calculated using t-tests for pairwise comparison with SAS software (SAS Institute Inc.).*

#### *PvCesA6*-RNAi Lines

Two transgenic lines (2 and 12) had shorter and fewer tillers (line 12 only) with smaller plant width compared with non-transgenic controls. These same two transgenic lines (2 and 12) also had significantly less dry biomass (68% for line 2, 77% for line 12) relative to non-transgenic controls (**Table 2B**) and were congruent with the highest levels of transcript reduction of *PvCesA6* (**Figure 4D**).

### Cell Wall Chemical Composition of Transgenic Plants

#### *PvCesA4*-Overexpressing Lines

The lignin content was unchanged in transgenic lines with the exception of a significant decrease (4%) in transgenic line 7 and an increase (8%) in transgenic line 10 compared with the non-transgenic controls. There were no significant differences between the S/G ratios of the transgenic and non-transgenic control lines. Cellulose content was decreased (6–33%) and xylan content was increased (3–12%) in the transgenic lines compared with the non-transgenic controls. Galactan and arabinan contents were decreased in all transgenic lines by 17–25% and 38–48%, respectively (**Table 3A**). Complete chemical composition data is presented in Supplementary Table S2A.

**Table 3:** Cell wall chemical composition of transgenic lines overexpressing *PvCesA4* or *PvCesA6* and non-transgenic (WT) controls.

A. *PvCesA4*-overexpression						
Lines	1	7	8	9	10	WT
Lignin	16.9 ± 0.1	**16.5 ± 0.2^∗^**	17.1 ± 0.1	17.4 ± 0.2	**18.5 ± 0.1^∗^**	17.2 ± 0.1
S/G	0.7 ± 0.01	0.7 ± 0.01	0.7 ± 0.02	0.7 ± 0.03	0.7 ± 0.01	0.7 ± 0.01
Cellulose	**28.1 ± 0.2^∗^**	**28.0 ± 0.4^∗^**	**28.7 ± 0.2^∗^**	**24.9 ± 0.8^∗^**	**20.4 ± 0.2^∗^**	30.5 ± 0.1
Hemicellulose	**20.8 ± 0.2^∗^**	**21.0 ± 0.2^∗^**	**20.7 ± 0.1^∗^**	**21.5 ± 0.4^∗^**	**23.4 ± 0.3^∗^**	22.9 ± 0.1
Xylan	17.7 ± 0.2	17.8 ± 0.2	17.7 ± 0.1	**18.2 ± 0.4^∗^**	**19.9 ± 0.2^∗^**	17.7 ± 0.1
Galactan	**0.9 ± 0.0^∗^**	**0.9 ± 0.0^∗^**	**0.9 ± 0.0^∗^**	**1.0 ± 0.0^∗^**	**1.0 ± 0.0^∗^**	1.2 ± 0.0
Arabinan	**2.2 ± 0.0^∗^**	**2.3 ± 0.0^∗^**	**2.1 ± 0.0^∗^**	**2.3 ± 0.0^∗^**	**2.5 ± 0.0^∗^**	4.0 ± 0.0

**B. *PvCesA6*-overexpression**						
**Lines**	**2**	**6**	**10**	**13**	**16**	**WT**

Lignin	**16.1 ± 0.1^∗^**	**16.0 ± 0.1^∗^**	**16.0 ± 0.2^∗^**	**16.3 ± 0.0^∗^**	**16.5 ± 0.2^∗^**	17.2 ± 0.1
S/G	**0.6 ± 0.01^∗^**	**0.6 ± 0.01^∗^**	**0.6 ± 0.02^∗^**	**0.6 ± 0.01^∗^**	**0.6 ± 0.01^∗^**	0.7 ± 0.01
Cellulose	**27.2 ± 0.3^∗^**	**28.2 ± 0.1^∗^**	**27.8 ± 0.3^∗^**	**28.1 ± 0.1^∗^**	**26.6 ± 0.1^∗^**	30.5 ± 0.1
Hemicellulose	**23.7 ± 0.3^∗^**	23.2 ± 0.1	**23.4 ± 0.0^∗^**	23.3 ± 0.1	22.8 ± 0.1	22.9 ± 0.1
Xylan	**18.2 ± 0.3^∗^**	18.0 ± 0.1	**18.1 ± 0.1^∗^**	**18.3 ± 0.1^∗^**	17.7 ± 0.1	17.7 ± 0.1
Galactan	1.2 ± 0.0	1.1 ± 0.0	1.1 ± 0.0	1.1 ± 0.0	1.1 ± 0.0	1.2 ± 0.0
Arabinan	**4.3 ± 0.0^∗^**	4.1 ± 0.0	**4.2 ± 0.1^∗^**	3.9 ± 0.0	4.0 ± 0.0	4.0 ± 0.0

*Values (weight% of cell wall residue) represent the mean of three biological replicates ± standard error. Bold values with asterisks are significantly different from controls at P ≤ 0.05^∗^ and P ≤ 0.01^∗∗^ as calculated using t-tests for pairwise comparison with SAS software (SAS Institute Inc.).*

#### *PvCesA6*-Overexpressing Lines

Lignin content was decreased significantly in all transgenic lines by 4–7% compared with the non-transgenic controls. There was a significant decrease in S/G ratio by 14% in all transgenic lines. Transgenic lines had decreased cellulose content (8–13%) and increased xylan content (2–4%) compared with the non-transgenic controls. There were no significant differences in galactan content between the transgenic and non-transgenic control lines, whereas arabinan content was increased in transgenic line-2 (8%) and line-10 (5%) (**Table 3B**). Complete chemical composition data is presented in Supplementary Table S2B.

#### *PvCesA4*-RNAi Lines

Lignin content was unchanged in the transgenic lines compared with the non-transgenic controls. There were no significant differences between the S/G ratio of the transgenic and non-transgenic control lines. Cellulose content was decreased (up to 9%) and xylan content was increased (up to 4%) in transgenic lines compared with the non-transgenic controls. There was no change in galactan content, whereas arabinan content was decreased up to 30% in transgenic lines compared with non-transgenic controls (**Table 4A**). Complete chemical composition data is presented in Supplementary Table S3A.

**Table 4:** Cell wall chemical composition of *PvCesA4*-RNAi or *PvCesA6*-RNAi transgenic lines and non-transgenic (WT) controls.

A. *PvCesA4*-RNAi				
Lines	12	15	20	WT
Lignin	16.9 ± 0.1	16.5 ± 0.1	16.6 ± 0.1	16.8 ±0.1
S/G	0.6 ± 0.01	0.6 ± 0.03	0.6 ± 0.02	0.6 ± 0.01
Cellulose	**30.1 ± 0.1^∗^**	**28.7 ± 0.1^∗^**	**29.4 ± 0.1^∗^**	31.4 ± 0.3
Hemicellulose	21.4 ± 0.2	21.5 ± 0.1	21.3 ± 0.2	21.4 ± 0.2
Xylan	**18.8 ± 0.1^∗^**	**18.5 ± 0.1^∗^**	18.0 ± 0.1	18.0 ± 0.2
Galactan	1.0 ± 0.0	1.0 ± 0.0	1.1 ± 0.0	1.1 ± 0.0
Arabinan	**1.6 ± 0.1^∗^**	**2.0 ± 0.1^∗^**	2.2 ± 0.0	2.3 ± 0.1

**B. *PvCesA6*-RNAi**				
**Lines**	**2**	**9**	**12**	**WT**

Lignin	16.5 ± 0.5	16.3 ± 0.1	16.2 ± 0.5	16.8 ± 0.1
S/G	0.6 ± 0.02	0.6 ± 0.02	0.6 ± 0.01	0.6 ± 0.01
Cellulose	**29.6 ± 0.4^∗^**	**28.9 ± 0.4^∗^**	**28.9 ± 0.4^∗^**	31.4 ± 0.3
Hemicellulose	22.0 ± 0.3	21.3 ± 0.3	21.1 ± 0.5	21.4 ± 0.2
Xylan	**18.7 ± 0.2^∗^**	**18.3 ± 0.3^∗^**	**18.5 ± 0.3^∗^**	18.0 ± 0.2
Galactan	1.2 ± 0.0	1.1 ± 0.0	1.1 ± 0.0	1.1 ± 0.0
Arabinan	2.1 ± 0.1	**1.9 ± 0.1^∗^**	**1.5 ± 0.2^∗^**	2.3 ± 0.1

*Values (weight% of cell wall residue) represent the mean of three biological replicates ± standard error. Bold values with asterisks are significantly different from controls at P ≤ 0.05^∗^ and P ≤ 0.01^∗∗^ as calculated using t-tests for pairwise comparison with SAS software (SAS Institute Inc.).*

#### *PvCesA6*-RNAi Lines

Lignin content was unchanged in the transgenic lines compared with the non-transgenic controls. There were no significant differences between the S/G ratio of the transgenic and non-transgenic control lines. Cellulose content was decreased (up to 8%) and xylan content was increased (up to 4%) in transgenic lines compared with non-transgenic controls. There was no change in galactan content, whereas arabinan content was decreased up to 35% in transgenic lines compared with non-transgenic controls (**Table 4B**). Complete chemical composition data is presented in Supplementary Table S3B.

### Sugar Release Efficiency of Transgenic Plants

#### *PvCesA4*-Overexpressing Lines

**FIGURE 5:**
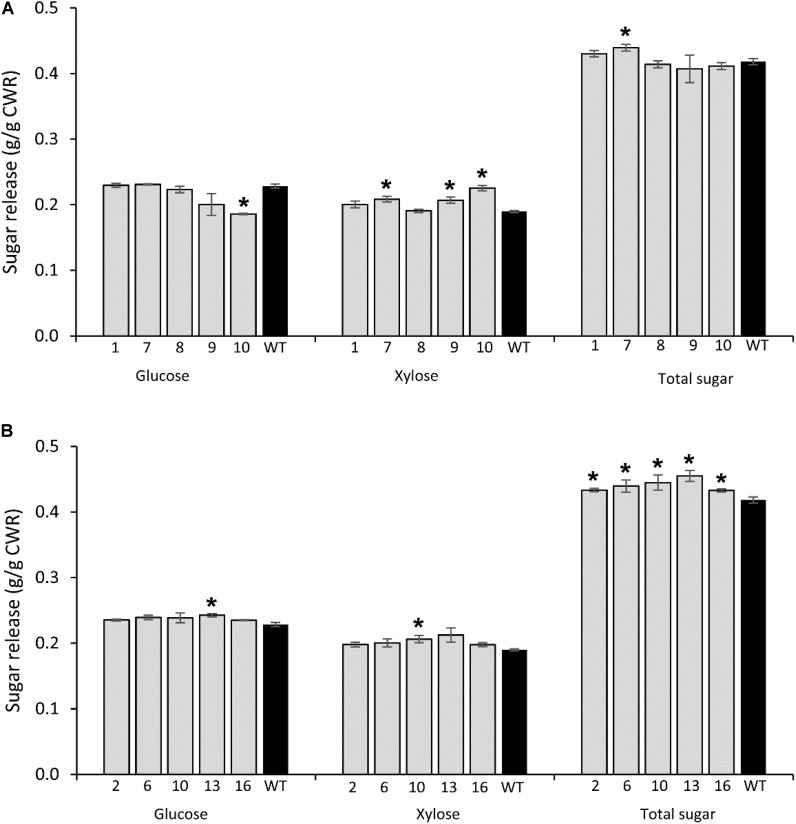
Glucose, xylose, and total sugar release by enzymatic hydrolysis from transgenic lines overexpressing *PvCesA4* **(A)** and *PvCesA6* **(B)** and non-transgenic (WT) controls. Bars represent mean values of three biological replicates ± standard error. Bars with asterisk are significantly different from controls at *P* ≤ 0.05^∗^ and *P* ≤ 0.01^∗∗^ as calculated using *t*-tests for pairwise comparison with SAS software (SAS Institute Inc.). CWR: cell wall residue.

Glucose release was significantly decreased in transgenic line 10 (19%), whereas xylose release was increased in transgenic line 7 (10%), line 9 (9%), and line 10 (19%) compared with the non-transgenic controls. No significant differences were observed in total sugar release between transgenic and non-transgenic control lines with the exception of an increase in transgenic line 7 (5%) (**Figure 5A**), which was congruent with the significant reduced lignin content (**Table 3A**).

#### *PvCesA6*-Overexpressing Lines

Glucose release was significantly increased in transgenic line 13 (7%), whereas xylose release was increased in line 10 (8%) compared with non-transgenic control. However, all transgenic lines had increased total sugar release by 4–9% compared with the non-transgenic controls (**Figure 5B**) and were congruent with the significant reduction in lignin content and S/G ratio (**Table 3B**).

#### *PvCesA4*-RNAi and *PvCesA6*-RNAi Lines

**FIGURE 6:**
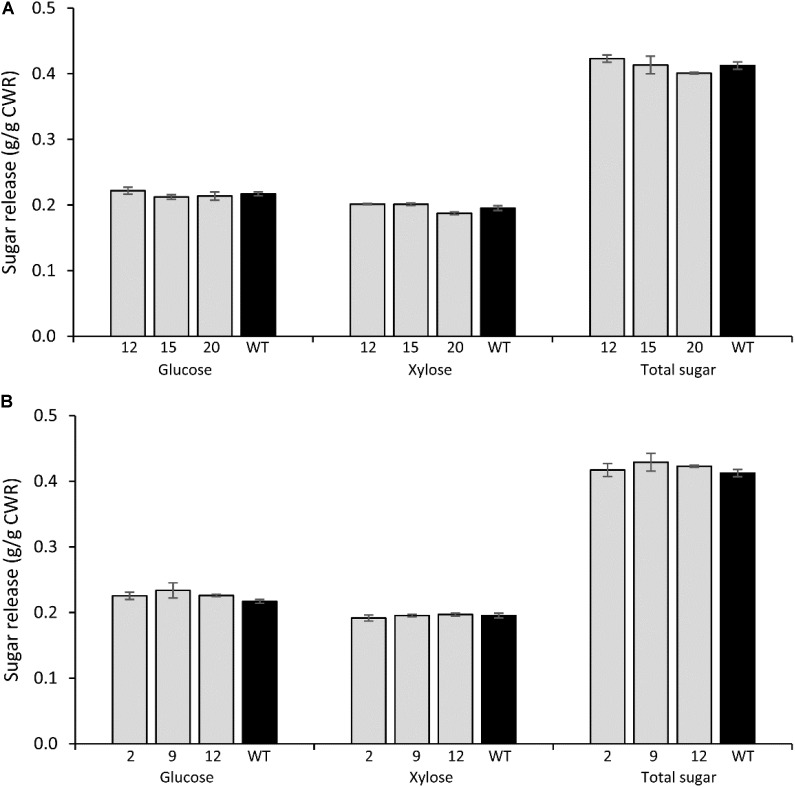
Glucose, xylose, and total sugar release by enzymatic hydrolysis from *PvCesA4*-RNAi **(A)** and *PvCesA6*-RNAi **(B)** transgenic lines and non-transgenic (WT) controls. Bars represent mean values of three biological replicates ± standard error. Bars with asterisk are significantly different from controls at *P* ≤ 0.05^∗^ and *P* ≤ 0.01^∗∗^ using *t*-tests for pairwise comparison with SAS software (SAS Institute Inc.). CWR: cell wall residue.

There were no significant differences in glucose, xylose, and total sugar release between the *PvCesA4*-RNAi or *PvCesA6*-RNAi transgenic lines and non-transgenic controls (**Figures 6A,B**).

### Cellulose Crystallinity of Transgenic Plants

#### *PvCesA4*-Overexpressing Lines

**FIGURE 7:**
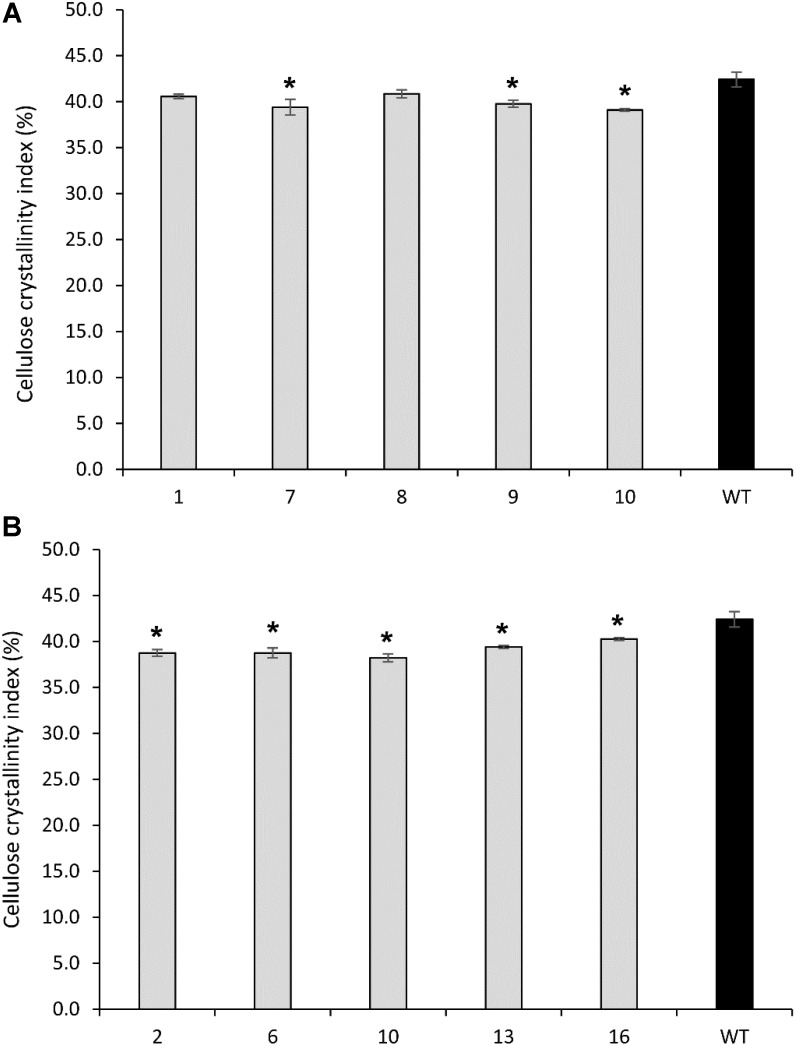
Cellulose crystallinity of cellulose extracted from transgenic lines overexpressing *PvCesA4* **(A)** and *PvCesA6* **(B)** and non-transgenic (WT) controls. Bars represent mean values of three biological replicates ± standard error. Bars with asterisk are significantly different from controls at *P* ≤ 0.05^∗^ and *P* ≤ 0.01^∗∗^ as calculated using *t*-tests for pairwise comparison with SAS software (SAS Institute Inc.).

Cellulose crystallinity was significantly decreased in transgenic line 7 (7%), line 9 (6%), and line 10 (8%) compared with the non-transgenic controls (**Figure 7A**).

#### *PvCesA6*-Overexpressing Lines

All transgenic lines showed a significant decrease in cellulose crystallinity by 5–10% compared with the non-transgenic controls (**Figure 7B**).

#### *PvCesA4*-RNAi and *PvCesA6*-RNAi Lines

**FIGURE 8:**
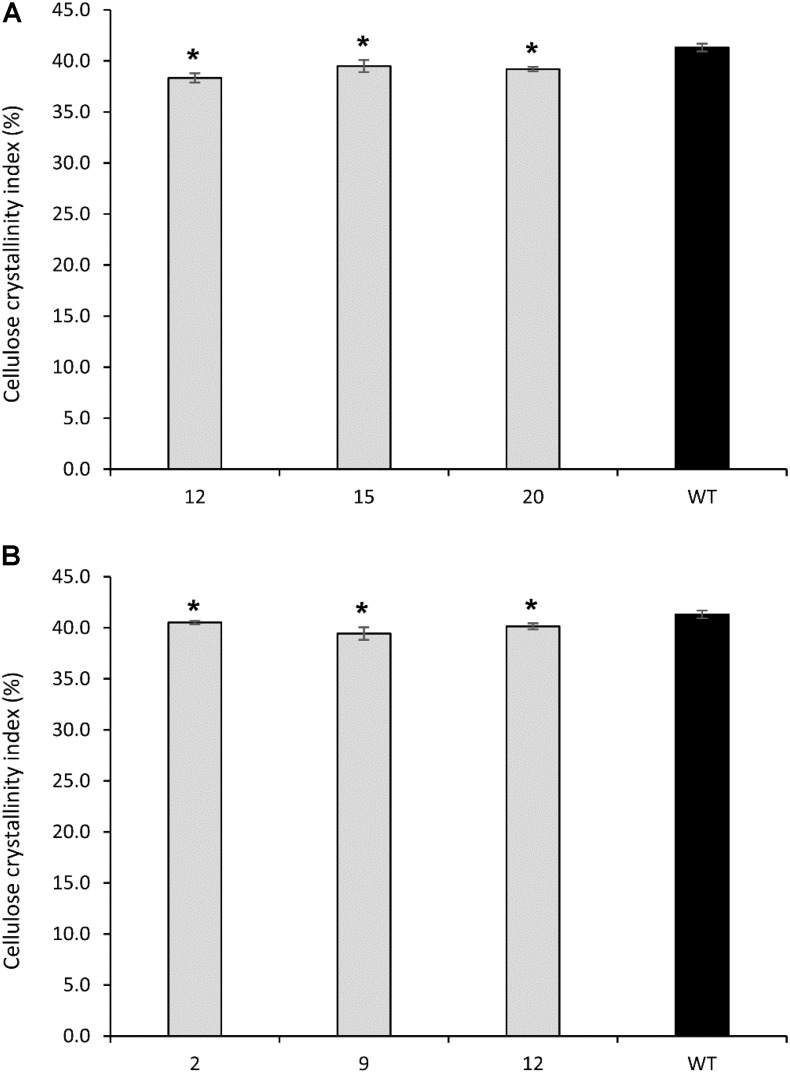
Cellulose crystallinity of cellulose extracted from *PvCesA4*-RNAi **(A)** and *PvCesA6*-RNAi **(B)** transgenic and non-transgenic (WT) lines. Bars represent mean values of three biological replicates ± standard error. Bars with asterisk are significantly different from controls at *P* ≤ 0.05^∗^ and *P* ≤ 0.01^∗∗^ as calculated using *t*-tests for pairwise comparison with SAS software (SAS Institute Inc.).

All *PvCesA4*-RNAi and *PvCesA6*-RNAi transgenic lines showed significantly decreased cellulose crystallinity up to 5 and 7%, respectively, compared with non-transgenic controls (**Figures 8A,B**).

### Cellulose Characteristics of Transgenic Plants

Cellulose degree of polymerization and polydispersity were unchanged in the *PvCesA4* and *PvCesA6* overexpressing lines and in the RNAi lines compared with their respective non-transgenic controls (Supplementary Tables S4, S5).

### Expression Level of Xylan Biosynthetic Genes *IRX9* and *IRX14* in Transgenic Plants Overexpressing *PvCesA4* and *PvCesA6*

Transcript abundance of *IRX9* and *IRX14* shown to be involved in xylan biosynthesis in Arabidopsis (Scheller and Ulvskov, 2010) was determined in *PvCesA4* and *PvCesA6* overexpressing lines. Expression analysis showed that *PvIRX9* transcripts were increased in the *PvCesA4* and *PvCesA6* transgenic lines, whereas *PvIRX14* transcripts were not affected as compared to those found in non-transgenic control (Supplementary Figure S6).

## Discussion

Understanding the enzymes responsible for the synthesis and regulation of cellulose synthesis is a key to the engineering of biofuel crops for cellulose production and more efficient extraction of glucose from cellulose. With a view to study the CesA machinery in switchgrass, we identified switchgrass *PvCesA4* and *PvCesA6* that have orthologues with other plant species. For example, PvCesA6 falls into the same clade as Arabidopsis AtCes2, AtCes5, AtCes6, AtCes9, and rice OsCesA3, OsCesA5, and OsCesA6. These proteins are all known to participate in primary wall cellulose synthesis (Wang et al., 2010; Endler and Persson, 2011). PvCesA4 falls into the same clade as Arabidopsis AtCes4 and AtCes8, and rice OsCesA4 and OsCesA7. These are known to be essential isoforms for secondary wall cellulose synthesis (Wang et al., 2010; Endler and Persson, 2011). Taken together, we conclude that PvCesA6 plays a role in primary cell wall formation and PvCesA4 in secondary cell wall formation in switchgrass.

Transcript expression analysis revealed that *PvCesA4* and *PvCesA6* are expressed in all the tissues tested, but that *PvCesA4* expression was highest in stems whereas *PvCesA6* expression was highest in leaves. Stem tissue is composed predominately of secondary cell walls that contain high amounts of cellulose and lignin which are valuable for biomass applications (Li F. et al., 2017). Furthermore, secondary cell walls of stems provide much of the rigidity and tensile and compression strength needed to support leaves and flowers, in contrast to the flexible primary wall of organs such as leaves (Fagard et al., 2000a). Consistently, higher secondary cell wall *CesA* gene expression in stems relative to that of primary cell wall *CesA* genes was observed in other plant species (Kotake et al., 2011; Li A. et al., 2013; Song et al., 2013; Mokshina et al., 2014; Chantreau et al., 2015; Petrik et al., 2016). A recent study involving switchgrass cell suspension cultures also showed a higher expression of *CesA4* associated with the secondary cell wall formation (Rao et al., 2017). Our results are congruent with the function of *PvCesA4* in secondary wall formation and *PvCesA6* in primary cell wall formation in switchgrass.

There was a negative association between the level of increasing *PvCesA4* and *PvCesA6* expression and plant biomass production. For example, *PvCesA4* (lines 8 and 9) and *PvCesA6* (lines 6 and 13) overexpression lines with the greatest transcript levels (up to 41-fold increase of the transgene) had the greatest decrease (up to 81%) in biomass yield. Conversely, there was a positive association between the level of decreasing *PvCesA4* and *PvCesA6* expression and plant biomass production. For example, the two transgenic RNAi-knockdown *PvCesA6* (lines 2 and 12) with the greatest reduction (up to 64%) in expression of the gene had up to 77% decreased biomass yield. In general, the reduction in plant biomass was associated with decreased plant height and width. Yet, increasing or decreasing *PvCesA4* and *PvCesA6* expression at low to moderate levels in the transgenic lines resulted in biomass production equivalent to that of the non-transgenic controls. Despite interest in the CesA family genes as potential targets for improving sugar yield in plant biomass, efforts to genetically manipulate members of the CesA gene family to achieve this goal have been challenging (Burton and Fincher, 2014). For example, overexpression of *CesA* genes in barley and poplar largely resulted in the silencing of both the transgenes and the endogenous genes (Joshi et al., 2011; Tan et al., 2015). Furthermore, although *CesAs* have been shown to be essential for plant growth (Persson et al., 2007), the overexpression of *CesA* genes has not generally led to improved plant growth but rather resulted in defective plant growth and reduced biomass yield in Arabidopsis, barley, and poplar plants (Zhong et al., 2003; Joshi et al., 2011; Tan et al., 2015). However, a recent study has demonstrated that overexpression of certain primary wall CesA6-like genes can improve plant growth in Arabidopsis (Hu H. et al., 2018). In the present study, we also observed that overexpression of *PvCesA6* (i.e., a putative primary wall CesA6-like gene) by moderate levels of transgene expression (line 2 with seven-fold increase) resulted in increased plant biomass largely by increasing plant tiller number, which may provide a useful trait for biomass crops such as switchgrass. Decreasing *CesA* expression in tobacco and flax by gene knockdown via the VIGS system and in *Brachypodium* by gene knockdown using an artificial microRNA system resulted in markedly shorter plants (Burton et al., 2000; Handakumbura et al., 2013; Chantreau et al., 2015). Studies of Arabidopsis and rice mutants impaired in *CesA* expression also reported severe growth inhibition of plants (Fagard et al., 2000b; Ellis et al., 2002; Tanaka et al., 2003; Taylor et al., 2003; Zhong et al., 2003; Wang et al., 2006; Zhang et al., 2009; Pysh et al., 2012; Rubio-Díaz et al., 2012; Wang et al., 2012). In contrast, however, rice mutants with amino acid alterations in CesA showed normal plant growth (Song et al., 2013) or greater biomass production (Li F. et al., 2017). In the present study, plant growth in the RNAi-knockdown *PvCesA* lines mostly depended on the level of reduction of transcript abundance of *PvCesA* where decreased expression at high levels (up to 64%) resulted in smaller plants attributing to the up to 77% decrease in biomass production, whereas low to moderate levels of decrease (11–36%) had no effect on plant growth and biomass production. Consistent with these observations, several of the transgenic lines with more than 70% reduced *CesA* expression failed to reach the R1 growth stage (data not shown). These results suggest that an optimized level of expression of the *CesA* genes by an inducible or appropriate promoter may be required to produce transgenic plants with the desired growth and cellulose content.

Cell wall chemical analyses showed that both the upregulation and downregulation of *PvCesA* were associated with a decrease in cellulose content in the transgenic lines. Cellulose content was also reduced in VIGS *CesA*-silenced tobacco and flax (Burton et al., 2000; Chantreau et al., 2015) and in the Arabidopsis, rice, and barley mutants (Ellis et al., 2002; Tanaka et al., 2003; Taylor et al., 2003; Zhong et al., 2003; Chen et al., 2005; MacKinnon et al., 2006; Paredez et al., 2008; Zhang et al., 2009; Burton et al., 2010; Kotake et al., 2011; Harris et al., 2012; Rubio-Díaz et al., 2012; Song et al., 2013; Li F. et al., 2017). Our results involving the reduced cellulose content in the RNAi *PvCesA*-silenced plants are in line with those reported studies, which may further suggest that *PvCesAs* are functional orthologues of *CesA* genes in the other plant species. The reduced cellulose content was also observed in the *PvCesA*-overexpressing lines. Co-expression of at least three *CesA* genes is essential for cellulose biosynthesis (Somerville, 2006), thus, the individual overexpression of *PvCesA* possibly interferes with the machinery controlling cellulose biosynthesis in switchgrass. Consistent with this, reduced cellulose content was shown in *CesA*-overexpressing transgenic Arabidopsis, barley, and poplar plants (Zhong et al., 2003; Joshi et al., 2011; Tan et al., 2015). In contrast, however, there is a recent study demonstrating that the overexpression of specific individual primary wall CesA6-like genes results in increased cellulose content in Arabidopsis (Hu H. et al., 2018). In regard to the hemicellulose content (i.e., non-cellulosic cell wall polysaccharides), xylan was increased while galactan and arabinan were mostly reduced or unchanged in *PvCesA* overexpressing or RNAi knockdown lines. Xylan is a major hemicellulose in cell walls of mature tissues of grasses while galactan and arabinan are more abundant in woody plants (Girio et al., 2010). Our expression analysis involving *IRX9* and *IRX14*, shown to be involved in xylan biosynthesis in Arabidopsis (Scheller and Ulvskov, 2010), showed that while *PvIRX9* expression was increased in the *PvCesA4* and *PvCes6* transgenic lines, the expression of *PvIRX14* was unaffected. It has been shown that IRX14, rather than IRX9, is required for xylan backbone synthesis in primary cell walls of Arabidopsis (Mortimer et al., 2015). Our results may suggest differential functions of these *IRXs* genes in xylan extension in switchgrass. It may also suggest that the increase in xylan content in *PvCesA4* and *PvCes6* transgenic lines is regulated at a transcriptional level (if a part).

Xylan content was also shown to positively affect the enzymatic digestibility of biomass by reducing cellulose crystallinity (Li F. et al., 2013). Thus increased xylan content is considered as another possible means to enhance lignocellulose saccharification in bioenergy crops. It is possible that the increased xylan content in *PvCesA*-overexpressing or RNAi knockdown lines was a compensation response to decreased cellulose production. In other plant species, reduced cellulose content was also associated with an increase in the non-cellulosic cell wall-related sugars (Burton et al., 2000; Kotake et al., 2011; Song et al., 2013; Chantreau et al., 2015; Li F. et al., 2017). The present study further supports a connection between the cellular machinery controlling cellulose and hemicellulose biosynthesis in switchgrass.

Increasing or decreasing *PvCesA* expression resulted in reduced cellulose crystallinity in the transgenic lines. Cellulose crystallinity has been demonstrated as a factor that negatively impacts saccharification efficiency (Himmel et al., 2007; Harris et al., 2012; Zhang W. et al., 2013; Li F. et al., 2017). Cellulose concentration is positively associated with cellulose crystallinity, which is negatively associated with biomass saccharification in most plant species (Wang et al., 2016). Therefore, there may exist an upper limit to direct cellulose synthesis in cell walls. Where examined, the reduction of cellulose content in CesA mutants and CesA-overexpressed transgenic plants is consistently associated with reduced cellulose crystallinity (Wang et al., 2016). Increased xylan content also leads to reduced cellulose crystallinity (Li F. et al., 2013). Thus, the reduced cellulose crystallinity observed in the present study could be the result of decreased cellulose content and/or increased xylan content in the transgenic lines. Although we must note that there was not a consistent association between the decreased cellulose crystallinity and sugar release efficiency in *PvCesA* overexpressing or RNAi knockdown lines.

The accessibility of cellulose to enzymatic breakdown into fermentable sugars is also limited by the presence of lignin, and genetic modifications of lignin have been shown to enhance biomass saccharification (Chen and Dixon, 2007; Baxter et al., 2014, 2015; Bonawitz et al., 2014; Eudes et al., 2014; Wilkerson et al., 2014; Hu Z. et al., 2018). Notably, lignin content and S/G ratio were decreased only in *PvCesA6*-overexpressing lines that also had an accompanying increase in sugar release efficiency. Lignin content was shown to be unchanged in the CesA rice mutants (Kotake et al., 2011; Li F. et al., 2017) and increased in *CesA*-overexpressing Arabidopsis (Hu H. et al., 2018) plants. Decreased lignin content and modified composition in the *PvCesA6*-overexpressing lines reported here might suggest an impact of *PvCesA6* expression on lignin biosynthesis and reduced recalcitrance in switchgrass. A goal for the genetic engineering of plant cell walls for improved biofuel production is to develop feedstock with increased biomass saccharification properties without effecting plant growth and biomass accumulation (Abramson et al., 2010). In line with this goal, we have shown that lines with low-to-moderate *PvCesA6*-overexpression had decreased recalcitrance, increased sugar release, and that some lines also grew better than wild type.

## Conclusion

In conclusion, we have shown that genetic manipulation of *PvCesA* can affect cellulose, hemicellulose, and lignin content and cellulose crystallinity to result in improved biomass digestibility without negatively, and in some cases even positively, affecting plant growth. These results validate *PvCesA* as cellulose synthesis genes and provide further insights into the effects of specific over-expression and knockdown expression of CesA on cell wall content and plant growth in switchgrass. In addition, our results suggest a direct or indirect association between cellulose, xylan, and lignin expression. These results may suggest a possible role for cellulose-xylan-lignin polymer covalent or non–covalent physical interaction in switchgrass biomass. Therefore, further study of the *PvCesA4* and *PvCesA6 t*ransgenic plants to identify specific wall fractions that may contain cellulose-xylan-lignin polymer cross-links or strong association is expected to yield insight into the mechanisms involved in cellulose synthesis and cell wall architecture of plant cell walls in switchgrass. Switchgrass is among several high-biomass grass species. Certainly, key cell wall enzymes in these species play a role in the fast growth of their herbaceous biomass. It would be interesting to use switchgrass *CesA* genes in complementation studies of genomically-characterized grasses such as rice and *Brachypodium*, in which native CesA orthologues have been knocked out. One could imagine the simultaneous directed overexpression of switchgrass *CesA* genes in rice while knocking out the rice orthologues, both singularly and in combinations. Such an approach could further elucidate functionality and perhaps serve to engineer cereal crops for higher productivity.

## Author Contributions

MM designed the experiments, performed the expression studies, participated in plant phenotyping and preparation of plant samples for cell wall analysis, analyzed the data, and wrote the manuscript. HB performed plant phenotyping and statistical analysis, participated in preparation of plant samples for cell wall analysis and RNA isolation. ML, XM, YP, and AR performed cellulose analyses. AB and DM performed phylogenetic tree work and contributed to the conception of the study. KK and NL performed cell wall chemical analyses. WW assisted with designing gene specific primers. J-YZ and MU performed cloning of the target genes. GT, RS, and MD performed lignin and sugar release analyses. Z-YW produced the transgenic plants. CNS conceived of the study and its design, coordination and assisted with interpretation of results, and revisions to the manuscript. All authors contributed to text, data analysis, read, and approved the final manuscript.

## Conflict of Interest Statement

The authors declare that the research was conducted in the absence of any commercial or financial relationships that could be construed as a potential conflict of interest.

## Footnotes

1 https://phytozome.jgi.doe.gov/.
