# Supplementary material for: Functional Analysis of Cellulose Synthase CesA4 and CesA6 Genes in Switchgrass (Panicum virgatum) by Overexpression and RNAi-Mediated Gene Silencing
Source: Front Plant Sci. 2018 Aug 3;9:1114. doi: 10.3389/fpls.2018.01114 (PMC6088197; doi:10.3389/fpls.2018.01114)
Supplement: Supplementary file 1 [file Data_Sheet_1.pdf]

TCACTCCCCTCCTCCCGGCGCCGCCGCTGCTGCTCGCC**ATGGACGCCGGCGGCCTCGCGGCGGGGTCGCACATGCGGGGC**
GAGCTGCACGTCGTGCGCGGCCGCGACGCGGAGCCGGGCGCCGCCCGGAGCGCCGCGGCGGACGTGAGGACGTGCCGCGT
GTGCGGGGACGAGGTCGGGGCGCGGGAGGACGGGCAGCCCTTCGTGGCGTGCGCCGAGTGCGGCTTCCCCGTGTGCCGGC
CCTGCTACGAGTACGAGCGCAGCGACGGCACGCAGCGCTGCCCGCAGTGCAACACCCGCTACAAGCGCCAGAAAGGGTGC
CCGAGGGTGGAAGGGGACGAGGACGACGGCCCGGAGATGGACGACCTGGAGGAGGAGTTCCCCGCCGCCAAGAGCCCGGG
TAAGAAGCCCCACGAGCCCGTCGCCTTCGACGTCTACTCGGAGAACGGGGAGCAGCCGCCGCAGCGGTGGCGGACGGGAG
GCCAGACGCTCTCCTCCTTCACCGGAAGCGTGGCCGGGAAGGACCTGGAGGCCGAGAGGGAGATGGAGCCGGGAGCAATG
GAGTGGAAGGACCGCATCGACAAGTGGAAGACCAAGCAGGAGAAGAGGGGCAACAAGCTCAACCCCGACGACAGCGACGA
CGACGACGAAAAGAACGACGACGAGTACATGCTGCTGGCCGAGGCCCGGCAGCCTCTGTGGCGCAAGGTCCCGATCCCGT
CGAGCCAGATCAACCCGTACCGCATCGTCATCGTCCTCCGCCTGGTGGTGCTGTGCTTCTTCCTCAAGTTCCGGATCACG
ACGCCGGCGACGGACGCCGTGCCGCTGTGGCTGGCGTCCGTGGTGTGCGAGCTCTGGTTCGCGCTCTCGTGGATCCTGGA
CCAGCTCCCCAAGTGGGCCCCCGTGACCCGCGAGACGTACCTGGACCGGCTGGCGCTGCGGTACGACCGCGAGGGCGAGG
CGTGCCGGCTGGCCCCGATCGACTTCTTCGTGAGCACGGTGGACCCGCTCAAGGAGCCCCCCCTGATCACCGCCAACACC
GTGCTCTCCATCCTCGCCGTCGACTACCCCGTCGACCGCGCCAGCTGCTACGTCTCCGACGACGGCGCCTCCATGCTGCT
CTTCGACACGCTGTCGGAGACGGCCGAGTTCGCGCGGCGCTGGGTGCCCTTCTGCAAGAAGTTCGGCGTGGAGCCCCGCG
CGCCCGAGTTCTACTTCTCCCAGAAGATGGACTACCTCAAGGACAAGGTGCAGCCGACCTTCGTCAAGGAGCGGCGCGCC
ATGAAGCGCGAGTACGAGGAGTTCAAGGTGCGCGTCAACGCGCTGGTGGCCAAGGCGCAGAAGAAGCCCGAGGAGGGGTG
GGTGATGCAGGACGGCACGCCGTGGCCCGGGAACAACACGCGCGACCACCCGGGCATGATCCAGGTGTACCTGGGCAGCC
AGGGCGCGCTCGACGTGGAGGGCCGCGAGCTGCCGCGGCTGGTGTACGTGTCCCGGGAGAAGCGCCCCGGGTACAACCAC
CACAAGAAGGCGGGCGCCATGAACGCGCTGGTGCGGGTGTCGGCCGTGCTCACCAACGCGCCCTTCATCCTCAACCTCGA
CTGCGACCACTACGTGAACAACAGCAAGGCGGTGCGCGAGGCCATGTGCTTCCTCATGGACCCGCAGCTGGGGAGGAAGC
TCTGCTACGTCCAGTTCCCGCAGCGCTTCGACGGCATCGACCGCCACGACCGCTACGCCAACCGCAACGTCGTCTTCTTC
GACATCAACATGAAGGGGCTGGACGGCATCCAGGGCCCCGTCTACGTCGGCACCGGCTGCGTCTTCAACCGCCAGGCGCT
CTACGGCTACGACCCGCCGCGCCCGGAGAAGAGGCCCAAGATGACGTGCGACTGCTGGCCGTCCTGGTGCTGCTGCTGCT
GCTGCTTCGGCGGCGGCAGCAAGCACCAGCGCCGCGGCAAGAACAAGGGCGGTGGCGCCGACTCCGGCGCCGACGAGCCG
CGCCGCGGCCTGCTCGGCTTCTACAGGAAGCGCGGCGGCAAGAAGGACAAGCTAGGCGCCAAGAAGGGCGGCGCCGGGCT
CTACAGCAAGAAGCACCAGCAGCGCGCCGCGTTCGAGCTGGAGGAGATCGAGGAGGGCCTGGAGGGGTACGAGGAGCTGG
ACCGCTCCTCGCTCATGTCGCAGAAGGGCTTCGAGAAGCGCTTCGGCCAGTCGCCGGTGTTCATCGCCTCCACGCTCGTC
GAGGACGGCGGGCTCCCGCAGGGCGCCGCCGCCGACCCCGCCTCGCTCATCAAGGAGGCCATCCACGTCATCAGCTGCGG
CTACGAGGAGAAGACCGAGTGGGGCAAGGAGATCGGGTGGATCTACGGGTCCGTGACAGAGGACATCCTTACTGGGTTCA
AGATGCACTGCCGCGGGTGGAAGTCGGTGTACTGCACGCCGGCGCGGCCGGCGTTCAAGGGCTCGGCGCCGATCAACCTG
TCCGACCGTCTCCACCAGGTGCTGCGCTGGGCGCTGGGCTCCGTGGAGATCTTCATGAGCCGCCACTGCCCGCTCTGGTA
CGCCTACGGCGGCCGCCTCAAGTGGCTGGAGCGCTTCGCCTACACCAACACCATCGTCTACCCCTTCACCTCCATCCCGC
TCCTCGCCTACTGCACCATCCCCGCCGTCTGCCTCCTCACCGGCAAGTTCATCATCCCCACGCTGAACAACCTGGCCAGC
ATCTGGTTCATCGCCTTGTTCCTGTCCATCATCGCGACGGGCGTGCTGGAGCTGCGGTGGAGCGGGGTGAGCATCGAGGA
CTGGTGGCGCAACGAGCAGTTCTGGGTCATCGGCGGCGTCTCCGCGCACCTCTTCGCCGTGTTCCAGGGCCTGCTCAAGG
TGCTGGGCGGCGTGGACACCAACTTCACCGTCACCTCCAAGGCGGCCGCCGACGAGACGGACGCCTTCGGCGAGCTCTAC
CTCTTCAAGTGGACCACCCTGCTGGTGCCCCCCACCACGCTCATCATCATCAACATGGTCGGCATCGTGGCCGGCGTCTC
CGACGCCGTCAACAACGGCTATGGCTCCTGGGGCCCGCTCTTCGGCAAGCTCTTCTTCTCCTTCTGGGTCATCGTCCACC
TCTACCCCTTCCTCAAGGGGCTCATGGGGAGGCAGAACCGGACGCCCACCATCGTCGTGCTCTGGTCCATCCTCCTCGCC
TCCATCTTCTCGCTCGTCTGGGTTAGGATCGACCCCTTCATCCCCAAGGCCAAGGGCCCCATCCTCAAGCCGTGCGGAGT
CGAGTGCTGAATTGATCGAGCTCGATTGTTGAATGTCCGTATAGATCAGTTGCTTGCATGTACGGACGCGACAGCGCGTT
TGCGCATGTACTTTTGGACCACCAGGATTCTACTCATATTCGACTTATTCTTGTACAAGTTTTTTGTGATCGAGTGAGAC
AGAGTACGTGTTGGGGTTGTACGACAAAAATGGAGATAAAAGAAGACAGAACAGCCGCTCCAAATTGTGCATTCCATTTA
TGATTTATGGCCAGAACGT

Figure S1. Sequence of the *PvCesA4* cDNA fragment. The full-length coding sequence for use in the overexpression cassette is bolded. The sequence selected for use in the RNAi cassette is underlined and highlighted.

CCCTTACACCTCCCACCGACGCTGCCGCCGCTGGCACGCATTTCCCTCCTCGTCGTCTTCCTCTTCCTCGGCGCGGCGCG
GCGCGGAGCCTGGTGGAAACCGCGCGGATGAGGATCCGCTGCTAGAGCGAGAGGAGCAAGTATCCTGCGCCAGCGTCGGC
CGCGGCGGCGGCGGAGGGGGAGGAGGAGGAGGCG**ATGGAGGCGAGCGCCGGGCTGGTGGCCGGCTCCCACAACCGCAACG**
AGCTCGTCGTCATCCGCCGCGACGGCGATCCCGGGCCGAAGCCGCTGCGGCAGCCGAACGGGCAGGTGTGCCAGATTTGC
GGCGACGACGTCGGACTCGCCCCCGGCGGGGAGCCCTTCGTGGCCTGCAACGAGTGCGCCTTCCCCGTCTGCCGGGACTG
CTACGAGTACGAGCGCCGGGAGGGCACCCAGAACTGCCCCCAGTGCAAGACCCGCTACAAGCGCCTCAAGGGCTGCGCGC
GCGTGCCCGGGGATGAGGAAGAGGACGGCGTCGACGACCTGGAGAACGAGTTCAACTGGGACGGCAATGAGTCGCAGTAT
GGCGCCGAGTCACTCCACGGCCACATGACCTACGGCCGTGGAGGCGACCTTAACGGCGTCCAGCAGCCTTTCCAGCTGAA
CCCCAATGTTCCCCTCCTCACCAACGGCCAGATGGTGGATGACATCCCACCGGAGCAGCACGCGCTGGTCCCGTCTTTCA
TGGGCGGTGGGGGCAAGAGGATCCACCCACTTCCATACGCCGATCCCAGTTTACCTGTCCAACCAAGGTCCATGGACCCA
TCCAAGGATCTTGCTGCGTATGGATATGGTAGTGTTGCATGGAAGGAGAGGATGGAGAGCTGGAAGCAGAAGCAGGAGAG
GATGCACCAGATGAGGAATGATGCAGGTGGTGATGATGGTGGTGATGACGATCTTCCACTAATGGATGAAGCAAGACAAC
CACTGTCCAGGAAAATTCCAATTCCATCAAGCCAGATTAATCCATATAGGATGATTATCATTATTCGGCTTGTGGTTTTG
GGGTTCTTCTTCCACTATCGACTGATGCATCCAGTGAACGATGCATTTGCTTTGTGGCTCATATCTGTTATCTGTGAAAT
TTGGTTTGCCATGTCTTGGATCCTTGATCAATTCCCAAAATGGTTCCCTATTGAGAGAGAGACATACTTAGACCGGCTGT
CATTGAGGTTCGACAAGGAAGGCCAGCCATCTCAACTTGCTCCAATCGATTTCTTTGTCAGTACAGTCGATCCATTAAAG
GAACCTCCTTTGGTCACAGCAAATACTGTTCTATCTATCCTTGCGGTGGATTATCCAGTTGATAAGGTTTCTTGCTATGT
TTCTGATGATGGTGCTGCAATGCTAACATTTGAAGCGTTGTCTGAAACATCCGAATTTGCAAAGAAATGGGTTCCTTTCT
GCAAAAGGTTCAATATTGAACCTCGTGCTCCAGAGTGGTACTTCCAACAGAAAATAGACTACTTGAAAGACAAGGTGGCG
GCAAGCTTTGTTGGTGAGAGGAGAGCGATGAAGAGAGAGTATGAGGAATTCAAGGTCAGAATCAATGCCTTGGTTGCTAA
AGCCCAGAAAGTTCCTGAAGAAGGATGGACAATGCAAGATGGGACCCCCTGGCCTGGAAACAATGTTCGTGATCATCCTG
GAATGATTCAGGTCTTCCTTGGCCAAAGTGGAGGTCTTGATTGCGAGGGAAATGAGCTACCACGATTGGTTTATGTTTCA
AGAGAAAAACGACCTGGCTATAACCATCATAAGAAAGCTGGTGCTATGAATGCATTGGTCAGAGTCTCTGCTGTACTAAC
AAATGCTCCCTATTTGTTGAACTTGGATTGTGATCACTACATCAACAACAGTAAGGCTATAAAGGAAGCAATGTGTTTTA
TGATGGATCCTTTATTGGGAAAGAAGGTGTGCTATGTGCAGTTCCCTCAAAGATTTGATGGGATTGATCGTCATGACCGA
TATGCTAACAGGAATGTCGTCTTTTTTGATATCAACATGAAAGGTTTGGATGGTATTCAAGGCCCAATCTATGTCGGTAC
TGGATGTGTATTTAGAAGGCAGGCACTATATGGTTATGATGCCCCTAAATCAAAAAAGCCACCATCAAGGACTTGCAATT
GCTGGCCAAAGTGGTGCTTTTGCTGTTGCTGCTGTGGTAACAGAAAGCACAAGAAGAAGACTACCAAACCTAAATCAGAG
AAGAAGAAAAGATTACTATTTTTCAAGAAAGAAGAAAATCAATCCCCTGCATATACTCTTGGTGAGATTGATGAAGGTGC
TCCAGGTGCTGAAAATGAAAAGGCTGGTATTGTGAATCAACAAAAATTAGAAAAGAAATTTGGTCAGTCTTCTGTTTTTG
TCACGTCCACACTTCTTGAGAATGGTGGGACCTTGAAGAGTGCAAGTCCAGCTTCTCTTTTGAAAGAAGCCATACATGTC
ATTAGTTGTGGTTATGAAGACAAGACTGACTGGGGAAAAGAGATTGGCTGGATCTATGGATCAGTCACAGAAGATATTCT
AACTGGCTTCAAGATGCATTGTCATGGTTGGCGGTCCATTTACTGCATACCTAAACGACCTGCATTCAAAGGTTCTGCAC
CTCTGAATCTTTCAGATCGTCTTCACCAGGTTCTTCGGTGGGCTCTTGGGTCTATTGAAATTTTCTTCAGCAACCATTGC
CCTCTTTGGTATGGGTATGGTGGTGGTTTGAAATTTTTGGAAAGATTTTCCTACATCAACTCCATCGTATATCCTTGGAC
GTCTATTCCTCTCTTGGCTTATTGTACATTGCCTGCCATCTGTTTGCTGACAGGAAAATTTATCACTCCAGAGCTAAACA
ATGTTGCCAGCCTCTGGTTCATGTCACTTTTTATCTGCATCTTTGCTACAAGCATACTAGAAATGAGATGGAGTGGTGTT
GGCATTGATAATTGGTGGAGGAATGAGCAGTTTTGGGTCATTGGAGGTGTGTCCTCGCACCTTTTTGCTGTGTTCCAAGG
ACTTCTCAAGGTCATAGCTGGTGTGGATACAAGCTTCACTGTGACATCAAAGGGTGGTGACGATGAAGAGTTCTCAGAGC
TATATACATTCAAGTGGACAACCTTACTAATACCTCCAACCACTCTGCTCTTGTTGAACTTCATTGGTGTGGTTGCTGGT
GTTTCAAATGCTATCAACAACGGATATGAATCATGGGGCCCTCTATTCGGGAAGCTCTTCTTTGCATTCTGGGTGATTGT
CCATCTGTATCCGTTCCTGAAAGGTTTGGTTGGAAGGCAAAACAGGACACCAACAATTGTCATTGTCTGGTCCATTCTGC
TGGCTTCAATCTTCTCACTCCTTTGGGTCCGGATCGATCCTTTCCTAGCAAAAAATGATGGCCCGCTTCTTGAGGAGTGT
GGTTTGGATTGCAACTAGGAGGTCAGCATATCAGCTCCCCCAGCGCCGAATGCTTGAAAATAAAAACCATTTACAATGTC
CCCTCTGTAAATAAAAATCCATGGTGGTAGCTCAGCCTCATGCCTTCAGATATATACTGGGCAGAATGGAGGCTATGGCA
ATCCTTGTGCAGTTGGGCCGTGGAATAGAACATATGCAAAGTGTTCGATTGTTCAGCATTCTTTATTACTTGGGCGCAAA
ATTGATCGGGTGAGAGCCGAAGAGCAAGGTGTCTGATTCTGCACTGCTCCCGTGTACAAACTTGGGTCTGAATAAGGCAG
GCGGGGATGGTTCTGCCAGTGGAAAAGAGCAACATGCACAGTATTTATACGGCGGCTCGTGTATGCCTGCCCATTGAAGG
GCTTGTTCTTACATGTTCGTCTATACTAGAAAAACAGAATATTAGTGTTTAGTTCTGTAGTTAAAAGTGCGTAAACTGTA
AAGGCTCGTTTTTTTGTTCGTGTACTGTAATGGTGGCAGTTGTGACATGGTCATGTCATAGAAGAAATGTTGTAAGGATG
AATGTGACTCGATTACAAACAATATATTGCCGGGATCTCTGCAGTTCGTTCACCAT

Figure S2. Sequence of the *PvCesA6* cDNA fragment. The full-length coding sequence for use in the overexpression cassette is bolded. The sequence selected for use in the RNAi cassette is underlined and highlighted.

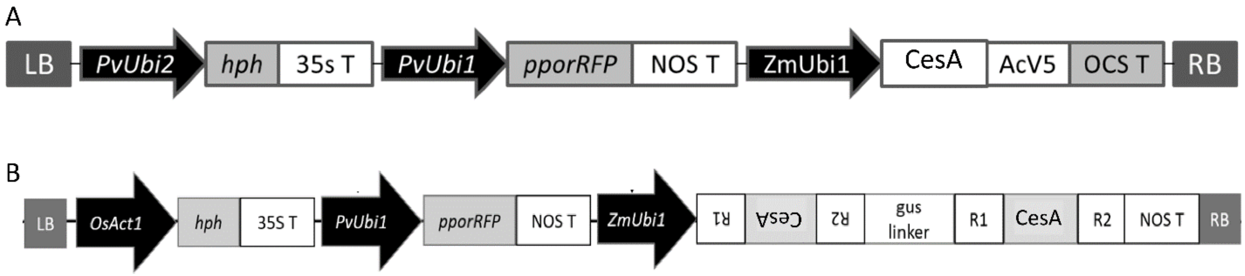

Figure S3. Schematic diagram of the pANIC10A plant overexpression vector (A) and pANIC8A plant RNAi-expression vector (B). Abbreviations: *PvUbi2* (switchgrass ubiquitin 2 promoter and intron), *hph* (hygromycin), 35S T (35S terminator), *PvUbi1* (switchgrass ubiquitin 1 promoter and intron), *pporRFP* (*Porites porites* red fluorescent protein), NOS T (nopaline synthase terminator), *ZmUbi1* (maize ubiquitin 1 promoter), *CesA* (*CesA* sequence fragment), AcV5 (epitope tag), OCS T (octopine synthase terminator), *OsAct1* (rice actin 1 promoter and intron), R1 and R2 (*attR1* and *attR2* recombinase sites), gus linker (linker from *uidA* gene), LB (left border), RB (right border).

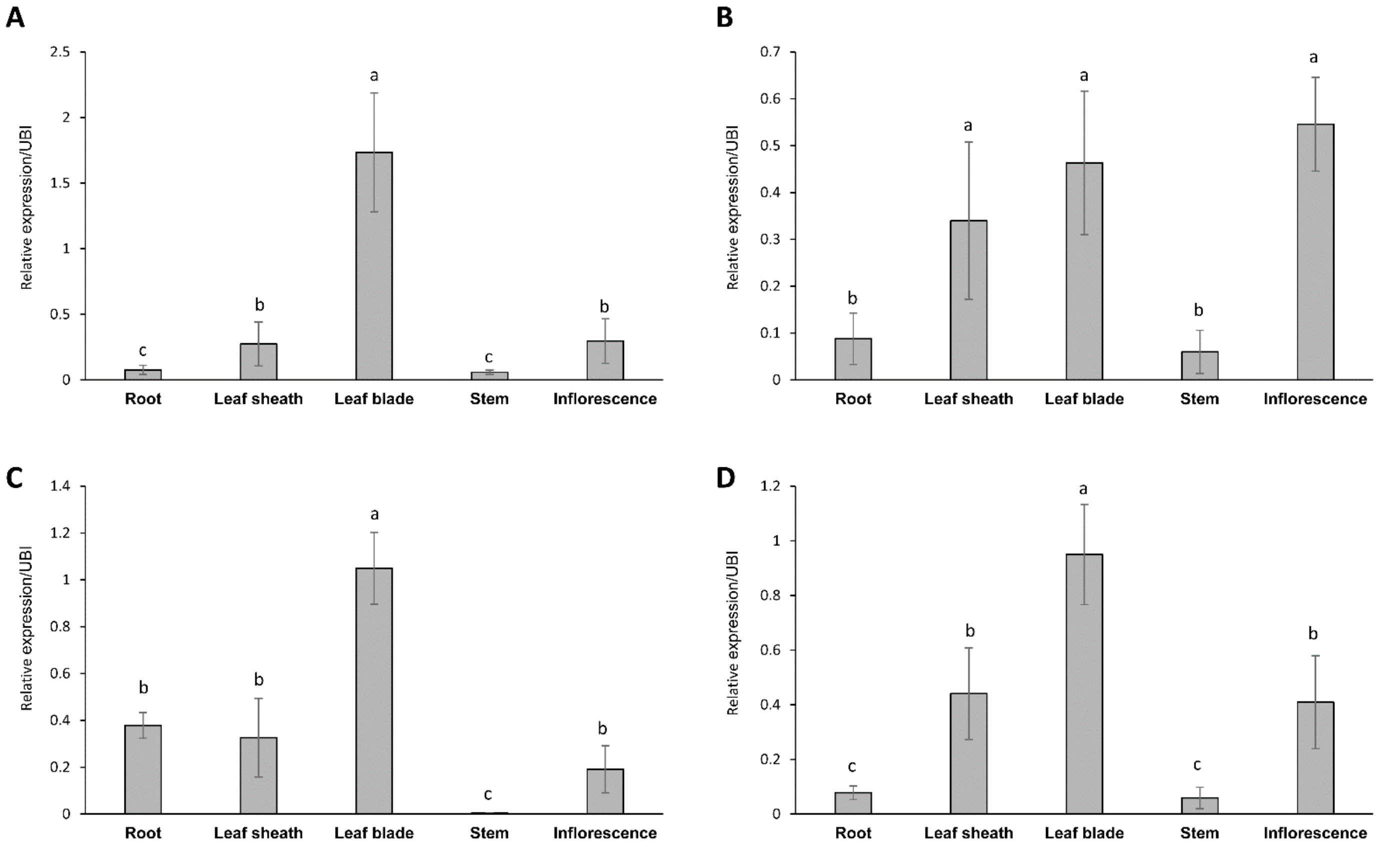

Figure S4. Expression patterns of *PvCesA6*-related genes in different plant tissues as determined by qRT-PCR. (A) Pavir.J27681, (B) Pavir.J01772, (C) Pavir.J26736, and (D) Pavir.J34300. Plant samples for RNA extraction used in the qRT-PCR experiments were collected at R1 (reproductive stage 1) developmental stage. The relative levels of transcripts were normalized to the switchgrass ubiquitin 1 gene expression (UBI). Bars represent mean values of three biological replicates ± standard error. Bars represented by different letters are significantly different at $P \leq 0.05$ as tested by LSD method with SAS software (SAS Institute Inc.)

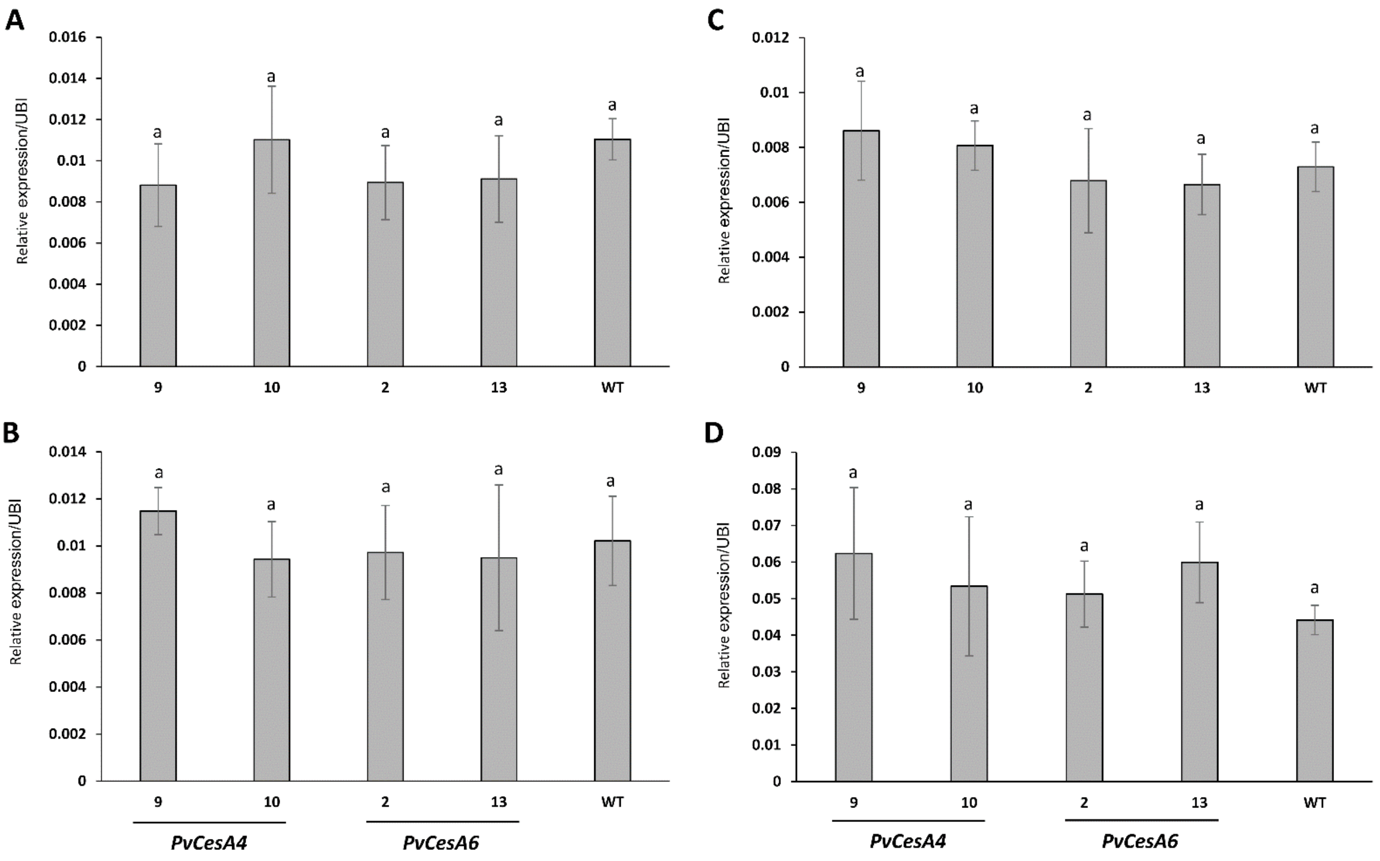

Figure S5. Relative transcript levels of other major secondary cell wall *PvCesA7* (A) and *PvCesA8* (B), and primary cell wall *PvCesA1* (C) and *PvCesA3* (D) in transgenic lines overexpressing *PvCesA4* and *PvCesA6* as determined by qRT-PCR. WT: non-transgenic control. The relative levels of transcripts were normalized to the switchgrass ubiquitin 1 gene expression (UBI). Bars represent mean values of three biological replicates ± standard error. Bars represented by different letters are significantly different at $P \leq 0.05$ as tested by LSD method with SAS software (SAS Institute Inc.).

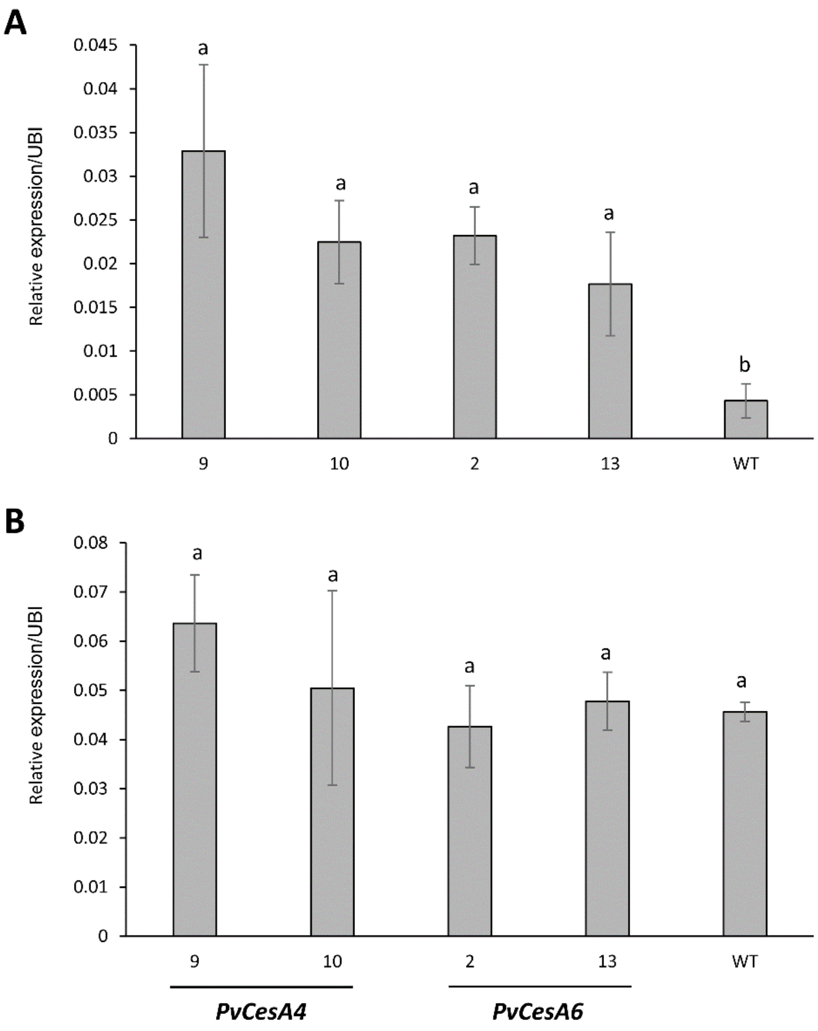

Figure S6. Relative transcript levels of *PvIRX9* (A) and *PvIRX14* (B) in transgenic lines overexpressing *PvCesA4* and *PvCesA6* as determined by qRT-PCR. WT: non-transgenic control. The relative levels of transcripts were normalized to the switchgrass ubiquitin 1 gene expression (UBI). Bars represent mean values of three biological replicates ± standard error. Bars represented by different letters are significantly different at $P \leq 0.05$ as tested by LSD method with SAS software (SAS Institute Inc.).

Table S1. List of primers used in this study.

Primer name	Primer sequence (5' – 3')
**Primers for cloning PvCesA6 (coding region)**	
560-oe-F	ATGGAGGCGAGCGCCGGGCTGG
560-oe-R	GTTGCAATCCAAACCACACTCCTC
**Primers for cloning PvCesA4 (coding region)**	
562-oe-F	CCGCTGCTGCTCGCCATGGA
562-oe-R	AATACAGCACTCGACTCCGCA
**Primers for cloning PvCesA6 (RNAi fragment)**	
561-kd-F	TCTGTAAATAAAAATCCATG
561-kd-R	ATGTAAGAACAAGCCCTTCA
**Primers for cloning PvCesA4 (RNAi fragment)**	
563-kd-F	AACAACGGCTATGGCTCC
563-kd-R	ACGGCTTGAGGATGGGGC
**qRT-PCR: Transgene specific primers for *PvCesA6***	
P5-F	CGCTTCTTGAGGAGTGTGGT
AcV5-R	ACCAGCCGCTCGCATCTTTC
**qRT-PCR: Transgene specific primers for *PvCesA4***	
P11-F	TCCATCCTCCTCGCCTCCATC
AcV5-R	ACCAGCCGCTCGCATCTTTC
**qRT-PCR: Gene specific primers for *PvCesA6* (endogenous)**	
P5-F	CGCTTCTTGAGGAGTGTGGT
P18-R	GCATGAGGCTGAGCTACCACC
**qRT-PCR: Gene specific primers for *PvCesA4* (endogenous)**	
P16-F	GCTCGTCTGGGTTAGGATCGAC
P13-R	GCGTCCGTACATGCAAGCAAC
**qRT-PCR: Primers for *PvUbi1***	
UBI-F	CAGCGAGGGCTCAATAATTCCA
UBI-R	TCTGGCGGACTACAATATCCA

qRT-PCR: Gene specific primers for Pavir.J27681 (*PvCesA6*-related) endogenous

P5-F	CGCTTCTTGAGGAGTGTGGT
681-R	GAAACAAGACATTTCATGTCTCTATC

qRT-PCR: Gene specific primers for Pavir.J01772 (*PvCesA6*-related) endogenous

772-F	CCTCTTGGAGGAGTGTGGTTT
772-R	AATATGGGCACAAAACATCTG

qRT-PCR: Gene specific primers for Pavir.J26736 (*PvCesA6*-related) endogenous

736-F	GAAATTTATTACGCCAGAGGTAAA
736-R	GCGAAGACACTCCTCCAATG

qRT-PCR: Gene specific primers for Pavir.J34300 (*PvCesA6*-related) endogenous

300-F	CTGGACTGCAACTGAGGAAG
300-R	CAGGATCTGTGTTTCTATCTACAGGA

qRT-PCR: Gene specific primers for Pavir.Ea00385 (*PvCesA1*) endogenous

385-F	CCTTTCATTTCACCTACACAGAA
385-R	CACGAAGTGGGGTAGACTTTG

qRT-PCR: Gene specific primers for Pavir.Ba03256 (*PvCesA3*) endogenous

256-F	GCTTCGATCTTCTCCTTGCT
256-R	CTCGAAAACACAATTCCTGG

qRT-PCR: Gene specific primers for Pavir.Bb02205 (*PvCesA7*) endogenous

205-F	CCTCCATCTTCTCCCTGCTC
205-R	ACAAGCTCCAGAATTTTTTGAATC

qRT-PCR: Gene specific primers for Pavir.Eb03139 (*PvCesA8*) endogenous

139-F	CCGAGCCTATCAACAGCAC
139-R	CCACATCTGCAACACCAGAA

qRT-PCR: Gene specific primers for Pavir.J27018 (*PvIRX9*) endogenous

IRX9-F	CGAGTGAGTACACGCCATTG
IRX9-R	GAACTAATCAAGCAAAATGAAAGC

qRT-PCR: Gene specific primers for Pavir.J37721 (*PvIRX14*) endogenous

IRX14-F	GGAAATTCGATCCTCCTCAG
IRX14-R	CCTGATTTGCTCTGACTTCTAC

Table S2. Cell wall chemical composition of transgenic lines overexpressing *PvCesA4* or *PvCesA6* and non-transgenic (WT) controls.

A. *PvCesA4*-overexpression

Lines	1	7	8	9	10	WT
Lignin	16.9±0.1	**16.5±0.2***	17.1±0.1	17.4±0.2	**18.5±0.1***	17.2±0.1
Acid Soluble Lignin	2.2±0.0	2.4±0.1	2.2±0.0	2.2±0.1	2.5±0.0	2.3±0.0
Acid Insoluble Lignin	14.7±0.1	**14.1±0.2***	14.9±0.1	15.2±0.1	**16.0±0.1***	14.9±0.2
Acetyl	**2.9±0.0***	**2.9±0.1***	**3.0±0.0***	**2.9±0.1***	**3.1±0.0***	2.5±0.0
Structural Ash	**2.6±0.1***	2.1±0.1	**2.4±0.1***	**2.5±0.1***	**2.5±0.1***	2.0±0.1
Extractives	22.9±0.2	**25.2±0.6***	**24.0±0.3***	**27.0±0.3***	**27.6±0.1***	22.9±0.2
Cellulose	**28.1±0.2***	**28.0±0.4***	**28.7±0.2***	**24.9±0.8***	**20.4±0.2***	30.5±0.1
Hemicellulose	**20.8±0.2***	**21.0±0.2***	**20.7±0.1***	**21.5±0.4***	**23.4±0.3***	22.9±0.1
Xylan	17.7±0.2	17.8±0.2	17.7±0.1	**18.2±0.4***	**19.9±0.2***	17.7±0.1
Galactan	**0.9±0.0***	**0.9±0.0***	**0.9±0.0***	**1.0±0.0***	**1.0±0.0***	1.2±0.0
Arabinan	**2.2±0.0***	**2.3±0.0***	**2.1±0.0***	**2.3±0.0***	**2.5±0.0***	4.0±0.0

B. *PvCesA6*-overexpression

Lines	2	6	10	13	16	WT
Lignin	**16.1 ±0.1***	**16.0±0.1***	**16.0±0.2***	**16.3±0.0***	**16.5±0.2***	17.2±0.1
Acid Soluble Lignin	2.4±0.0	**2.6±0.0***	2.5±0.1	2.4±0.0	2.5±0.1	2.3±0.0
Acid Insoluble Lignin	**13.7±0.1***	**13.4±0.1***	**13.5±0.2***	**13.9±0.0***	**14.0±0.2***	14.9±0.2
Acetyl	**2.8±0.0***	**2.7±0.0***	2.6±0.1	**2.8±0.0***	2.4±0.0	2.5±0.0
Structural Ash	**3.1±0.3***	**2.8±0.1***	**2.4±0.1***	**3.1±0.2***	**3.0±0.2***	2.0±0.1
Extractives	**26.4±0.6***	**24.7±0.3***	**24.8±0.3***	**24.1±0.1***	**26.0±0.1***	22.9±0.2
Cellulose	**27.2±0.3***	**28.2±0.1***	**27.8±0.3***	**28.1±0.1***	**26.6±0.1***	30.5±0.1
Hemicellulose	**23.7±0.3***	23.2±0.1	**23.4±0.0***	23.3±0.1	22.8±0.1	22.9±0.1
Xylan	**18.2±0.3***	18.0±0.1	**18.1±0.1***	**18.3±0.1***	17.7±0.1	17.7±0.1
Galactan	1.2±0.0	1.1±0.0	1.1±0.0	1.1±0.0	1.1±0.0	1.2±0.0
Arabinan	**4.3±0.0***	4.1±0.0	**4.2±0.1***	3.9±0.0	4.0±0.0	4.0±0.0

Values (weight% of cell wall residue) represent the mean of three biological replicates ± standard error. Bold values with asterisks are significantly different from controls at $P \leq 0.05$* and $P \leq 0.01$** as calculated using *t*-tests for pairwise comparison with SAS software (SAS Institute Inc.).

Table S3. Cell wall chemical composition of *PvCesA4*-RNAi or *PvCesA6*-RNAi transgenic lines and non-transgenic (WT) controls.

A. *PvCesA4*-RNAi

Lines	12	15	20	WT
Lignin	16.9±0.1	16.5±0.1	16.6±0.1	16.8±0.1
Acid Soluble Lignin	2.7±0.1	2.7±0.1	2.7±0.1	2.5±0.0
Acid Insoluble Lignin	14.2±0.1	13.8±0.2	13.9±0.1	14.3±0.1
Acetyl	3.1±0.1	3.0±0.1	2.9±0.1	2.9±0.1
Structural Ash	2.1±0.1	**2.5±0.1***	**2.7±0.1***	2.2±0.0
Extractives	**23.2±0.2***	**24.8±0.2***	**23.6±0.3***	22.0±0.3
Cellulose	**30.1±0.1***	**28.7±0.1***	**29.4±0.1***	31.4±0.3
Hemicellulose	21.5±0.2	21.5±0.1	21.3±0.2	21.4±0.2
Xylan	**18.8±0.1***	**18.5±0.1***	18.0±0.1	18.0±0.2
Galactan	1.0±0.0	1.0±0.0	1.1±0.0	1.1±0.0
Arabinan	**1.6±0.1***	**2.0±0.1***	2.2±0.0	2.3±0.1

B. *PvCesA6*-RNAi

Lines	2	9	12	WT
Lignin	16.4±0.5	16.3±0.1	16.2±0.5	16.8±0.1
Acid Soluble Lignin	2.7±0.1	2.6±0.1	2.7±0.1	2.5±0.0
Acid Insoluble Lignin	13.8±0.5	13.7±0.1	**13.5±0.1***	14.3±0.1
Acetyl	3.2±0.2	3.0±0.2	3.2±0.2	2.9±0.1
Structural Ash	**3.0±0.1***	**3.1±0.1***	**2.9±0.1***	2.2±0.0
Extractives	**23.8±0.1***	**24.5±0.1***	**23.9±0.1***	22.0±0.3
Cellulose	**29.6±0.4***	**28.9±0.4***	**28.9±0.4***	31.4±0.3
Hemicellulose	22.0±0.3	21.2±0.3	21.1±0.5	21.4±0.2
Xylan	**18.7±0.2***	**18.3±0.3***	**18.5±0.3***	18.0±0.2
Galactan	1.2±0.0	1.1±0.0	1.1±0.0	1.1±0.0
Arabinan	2.1±0.1	**1.9±0.1***	**1.5±0.2***	2.3±0.1

Values (weight% of cell wall residue) represent the mean of three biological replicates ± standard error. Bold values with asterisks are significantly different from controls at $P \leq 0.05$* and $P \leq 0.01$** as calculated using *t*-tests for pairwise comparison with SAS software (SAS Institute Inc.).

Table S4. Characteristics of cellulose extracted from transgenic lines overexpressing *PvCesA4* or *PvCesA6* and non-transgenic (WT) controls.

A. *PvCesA4*-overexpression

Lines	Degree of polymerization (DP_n)	Degree of polymerization (DP_w)	Polydispersity index (PDI)
1	894 ± 88	5983 ± 140	6.8 ± 0.47
7	723 ± 31	5799 ± 66.0	8.1 ± 0.40
8	599 ± 109	5398 ± 427	9.5 ± 1.22
9	571 ± 83	5091 ± 594	9.0 ± 0.36
10	572 ± 92	5339 ± 245	9.7 ± 1.23
WT	693 ± 31	5685 ± 236	8.3 ± 0.66

A. *PvCesA6*-overexpression

Lines	Degree of polymerization (DP_n)	Degree of polymerization (DP_w)	Polydispersity index (PDI)
2	724 ± 19	5956 ± 100	8.2 ± 0.17
6	624 ± 68	5948 ± 158	9.7 ± 0.76
10	632 ± 19	5820 ± 61	9.2 ± 0.23
13	527 ± 114	5823 ± 216	9.4 ± 0.64
16	555 ± 57	5311 ± 166	9.7 ± 0.63
WT	693 ± 31	5685 ± 236	8.3 ± 0.66

Values represent the mean of three biological replicates ± standard error. Number-average (DP_n) and weight-average (DP_w) degree of polymerization of cellulose. Values with asterisks are significantly different from controls at $P \leq 0.05$* and $P \leq 0.01$** as calculated using *t*-tests for pairwise comparison with SAS software (SAS Institute Inc.).

Table S5. Characteristics of cellulose extracted from *PvCesA4*-RNAi or *PvCesA6*-RNAi transgenic lines and non-transgenic (WT) controls.

A. *PvCesA4*-RNAi

Lines	Degree of polymerization (DP_n)	Degree of polymerization (DP_w)	Polydispersity index (PDI)
12	559 ± 67	5629 ± 92	10.3 ± 1.09
15	680 ± 22	5748 ± 111	8.5 ± 0.27
20	726 ± 62	5883 ± 223	8.2 ± 0.52
WT	701 ± 30	5754 ± 194	8.3 ± 0.64

B. *PvCesA6*-RNAi

Lines	Degree of polymerization (DP_n)	Degree of polymerization (DP_w)	Polydispersity index (PDI)
2	714 ± 42	5917 ± 20.0	8.4 ± 0.55
9	649 ± 96	5960 ± 125	9.6 ± 1.43
12	707 ± 5.0	5621 ± 251	8.0 ± 0.41
WT	701 ± 30	5754 ± 194	8.3 ± 0.64

Values represent the mean of three biological replicates ± standard error. Number-average (DP_n) and weight-average (DP_w) degree of polymerization of cellulose. Values with asterisks are significantly different from controls at $P \leq 0.05$* and $P \leq 0.01$** as calculated using *t*-tests for pairwise comparison with SAS software (SAS Institute Inc.).
